# ACOX2 destabilizes the MRE11-RAD50-NBS1 complex and boosts anticancer immunity via the cGAS-STING pathway in clear cell renal cell carcinoma

**DOI:** 10.1186/s12943-025-02420-9

**Published:** 2025-10-21

**Authors:** Shiqi Ye, Wenhao Xu, Zheqi Chen, Chuanying Zhu, Qintao Ge, Jiahe Lu, Kun Chang, Xi Tian, Aihetaimujiang Anwaier, Shuxuan Zhu, Siqi Zhou, Wei Zhang, Yue Wang, Jianyuan Zhao, Lingling Li, Yan Shi, Tingting Cai, Danfeng Xu, Xiangyu Zhou, Dingwei Ye, Hailiang Zhang

**Affiliations:** 1 Department of Urology, Huadong Hospital, Fudan University; Department of Urology, Fudan University Shanghai Cancer Center; Department of Oncology, Shanghai Medical College, Fudan University; Shanghai Genitourinary Cancer Institute, Fudan University, 200040 Shanghai, China; 2 Department of Urology, Ruijin Hospital, Shanghai Jiao Tong University School of Medicine, 200025 Shanghai, China; 3 State Key Laboratory of Genetic Engineering, School of Life Sciences, Human Phenome Institute, Fudan University, 200433 Shanghai, China; 4 Department of Oncology, Xin Hua Hospital Affiliated to Shanghai Jiao Tong University School of Medicine, 200092 Shanghai, China; 5 Institute for Developmental and Regenerative Cardiovascular Medicine, MOE-Shanghai Key Laboratory of Children’s Environmental Health, Xinhua Hospital, Shanghai Jiao Tong University School of Medicine, 200092 Shanghai, China; 6 Obstetrics & Gynecology Hospital of Fudan University, Shanghai Key Lab of Reproduction and Development, Shanghai Key Lab of Female Reproductive Endocrine Related Diseases, Fudan University, 200433 Shanghai, China

**Keywords:** ACOX2, MRE11-RAD50-NBS1 complex, Homologous recombination repair, cGAS-STING pathway, Tertiary lymphoid structures

## Abstract

The rapid development of ICI-based immunotherapy has ushered in a promising era for clear cell renal cell carcinoma (ccRCC). However, durable clinical responses remain limited to a subset of patients. Therefore, identifying novel predictive biomarkers and developing effective combination immunotherapies are critical for advancing personalized ccRCC management. In this study, we report that ccRCC patients exhibiting elevated ACOX2 expression may benefit from PARPi in combination with ICI. Multi-omics cohorts show ACOX2 is significantly downregulated in ccRCC and correlated with improved clinical prognosis. ACOX2 inhibits the growth of ccRCC both in vitro and in vivo. Mechanistically, ACOX2 interacts with MRE11 and inhibits the binding of MRE11 and RAD50, thereby destabilizing the MRE11-RAD50-NBS1 (MRN) complex. Furthermore, ACOX2 activates the cGAS-STING pathway, correlates with more mature tertiary lymphoid structures (TLS), and enhances CD8^+^ T cell infiltration and activity. Therapeutically, preclinical ccRCC models with high ACOX2 expression, including ccRCC cells, cell-derived xenograft (CDX), patient-derived organoid (PDO), patient-derived xenograft (PDX), and immunocompetent mouse models show increased sensitivity to PARPi plus anti-PD-1 therapy. In conclusion, our findings elucidate a pivotal role of ACOX2 in inhibiting HRR and propose that PARPi, either alone or in combination with anti-PD-1 therapy, represents a promising treatment strategy for ccRCC with elevated ACOX2 expression.

**Graphical Abstract:**

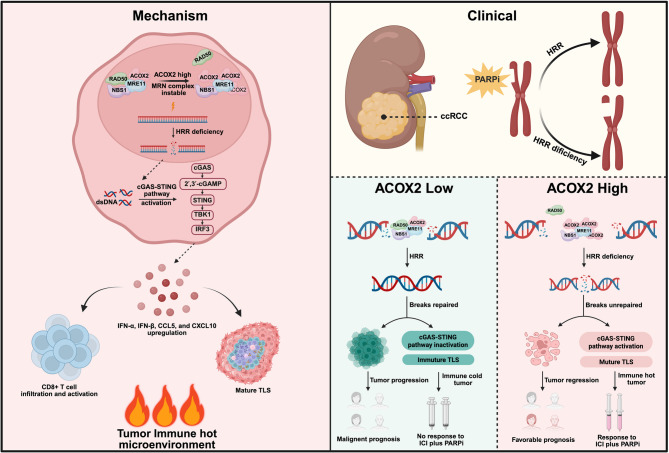

**Supplementary Information:**

The online version contains supplementary material available at 10.1186/s12943-025-02420-9.

## Introduction

Renal cell carcinoma (RCC) ranks among the top ten prevalent and fatal malignant tumors, with an estimated 431,288 new cases and 179,368 deaths globally in 2020 [1, 2]. Clear cell renal cell carcinoma (ccRCC), which accounts for approximately 80% of RCC cases, is an aggressive histological subtype originating from the proximal tubular epithelium [3, 4]. While early-stage ccRCC can be effectively managed through surgical interventions, up to one-third of patients present with or eventually develop metastatic disease [5]. Metastatic ccRCC (mccRCC) is almost universally lethal with an estimated five-year survival rate of around 15% [6]. The therapeutic landscape of mccRCC has undergone a significant revolution with the approval of immunotherapy agents, particularly immune checkpoint inhibitor (ICI) [7, 8]. However, a substantial number of patients still do not achieve durable responses, underscoring the urgent need for novel combination immunotherapy and predictive biomarkers for immunotherapy [9].

Increasing clinical studies are currently investigating combination therapies based on ICI to develop more effective therapeutic strategies for mccRCC [10]. Poly (ADP-ribose) polymerase (PARP) is a nuclear enzyme crucial for DNA repair [11]. PARP inhibitors (PARPi) are the first clinically approved drugs designed to exploit the synthetic-lethal strategy [12]. Typically, the initiating events for ccRCC tumorigenesis involve the loss of biallelic *von Hippel-Lindau (VHL)* gene and alterations in chromosome 3p [13]. In recent years, the etiology of DNA damage repair (DDR) defects has been increasingly recognized in ccRCC [14]. Moreover, accumulating evidence suggests that DDR defects may serve as potential predictive biomarkers for response to PARPi or ICI in mccRCC patients [9, 15, 16]. Consequently, exploring the underlying mechanisms of DDR defects in ccRCC is crucial for developing novel treatment strategies.

As the most dangerous types of DNA lesions, DNA double-strand breaks (DSBs) pose a significant threat to genome stability [17]. Two primary pathways dominate the repair of DSBs in somatic mammalian cells: homologous recombination repair (HRR) and non-homologous end joining (NHEJ) [18]. The MRE11–RAD50–NBS1 (MRN) complex acts as a pivotal sensor and responder to DNA damage, orchestrating HRR through DNA ends resection [19, 20]. MRE11, the core component of the MRN complex, possesses both endonuclease and exonuclease activities [21, 22]. RAD50 masters ATP hydrolytic activity to induce conformational change of the complex, which is essential for the MRE11-mediated nuclease activity at DSB sites [23, 24]. NBS1 functions as a flexible adaptor domain, delivering signals through binding to ATM or ATR [25, 26]. In recent years, the role of the MRN complex in tumorigenesis has garnered increasing attention [23, 27]. However, the precise regulatory mechanisms of the DNA repair machinery and their implications for cancer treatments remain to be fully elucidated [27].

A growing body of studies supports the dynamic interplay between DDR and antitumor immunity [28, 29]. The cyclic guanosine monophospha-adenosine monophosphate synthase-stimulator of interferon genes (cGAS-STING) pathway has generated considerable interest in immuno-oncology in recent years, especially in DDR-defective tumors. Defects in the DDR lead to excessive abnormal DNA leaking from the nucleus into the cytoplasm, which serves as a primary tumor cell-intrinsic mechanism to activate the cGAS-STING pathway [30, 31]. Subsequently, activated STING initiates downstream signaling that upregulates type I interferons (IFNs) and IFN-stimulated genes (ISGs), thereby regulating and reshaping the complex tumor immune microenvironment (TIME) [32]. However, the mechanisms by which chromatin regulators modulate DNA repair and their potential link to immunity remain unclear in ccRCC.

Tertiary lymphoid structures (TLS) are ectopic and organized lymphoid aggregates, which form in chronically inflamed environments, including cancer [33]. Our team previously proposed the definitions of TLS spatial heterogeneity (intratumoral and peritumoral TLS) and maturity heterogeneity, including early TLS (E-TLS), primary follicle-like TLS (PFL-TLS), and secondary follicle-like TLS (SFL-TLS), in ccRCC for the first time [34, 35]. Moreover, we reported that both intratumoral TLS and mature TLS (SFL-TLS) are associated with better clinical prognosis and immunotherapy response in ccRCC [34, 35]. As a powerful prognostic and predictive biomarker in various cancer types, such as melanoma, ovarian cancer, pancreatic ductal adenocarcinomas (PDAC), TLS may play a promising role in next-generation immunotherapy [36–38]. However, the mechanisms that trigger TLS formation in cancer and strategies to target TLS for improved anticancer efficacy remain poorly understood [39, 40]. Interestingly, recent studies have demonstrated that activation of the cGAS-STING pathway significantly enhances immune cell infiltration and the expression of lymphocyte-recruiting chemokine, thereby promoting TLS formation and elevating anticancer immunity in melanoma and lung carcinoma [41, 42].

Acyl-CoA oxidases (ACOXs), which catalyze the conversion of acyl-CoA to enoyl-CoA, are the key enzymes in peroxisomal fatty acid β-oxidation [43, 44]. To date, three distinct ACOXs (ACOX1, ACOX2, and ACOX3) have been identified, each exhibiting different substrate specificities [45]. Notably, ACOX2 is the only ACOX involved in bile acid biosynthesis and is responsible for the degradation of branched-chain fatty acids. ACOX2 is expressed in all organs, with predominant expression in the liver and kidney [46–48]. The amino acid sequence of ACOX2 is highly evolutionarily conserved across species, including *Homo sapiens*, *Caenorhabditis elegans*, *Solanum lycopersicum*, *Arabidopsis thaliana*, and *Saccharomyces cerevisiae*. Given that bile acid biosynthesis occurs exclusively in the liver and the contribution of branched-chain fatty acids degradation in peroxisome to overall fatty acid β-oxidation is minimal, ACOX2 may play a role in other essential biological processes [47, 49]. Recent studies have increasingly highlighted the significant role of ACOX2 in regulating tumorigenesis [50–53]including prostate cancer (PCa), non-small cell lung cancer (NSCLC), colorectal cancer (CRC), and breast cancer (BRCA) [50–53]. Interestingly, ACOX2 mediates tumorigenesis mostly through non-metabolic pathways in these contexts [50–53]. Our team previously reported that ACOX2 deficiency was associated with the progression of primary malignant cardiac tumor [54]. More importantly, we discovered that ACOX2 acted as a regulator of DDR, thus influencing the tumorigenesis of hepatocellular carcinoma (HCC) [47]. However, the precise role and molecular mechanism of ACOX2 in ccRCC have yet to be clarified.

In this study, we elucidate the anticancer role of ACOX2 in ccRCC for the first time. Mechanistically, ACOX2 interacts with MRE11, disrupting the binding of MRE11 and RAD50. This interference impairs the assembly of the MRN complex, thereby inhibiting HRR efficiency and aggravating DSBs accumulation. Furthermore, ACOX2 activates the cGAS-STING pathway and is associated with more mature TLS. Clinically, our findings indicate that PARPi, either alone or in combination with anti-PD-1 therapy, could be a promising treatment strategy for ccRCC patients exhibiting elevated ACOX2 expression.

## Methods and materials

### Sex as a biological variable

In animal experiments, a male to female mouse ratio of 2:1 was utilized to mimic the human context, where males are reported to have a twofold higher incidence of kidney cancer compared to females, along with a higher mortality rate [55]. Additionally, our investigation included the analysis of male and female ccRCC patients.

### Cell lines

Human embryonic kidney cell HEK293T, human renal cortex proximal tubule epithelial cell HK-2, and human RCC cell lines containing 786-O, 769-P, A-498, Caki-1, Caki-2, ACHN, and SW839 were obtained from ATCC, authenticated by STR profiling, and tested negative for mycoplasma contamination. HEK293T was cultured in DMEM medium (Gibco) supplemented with 10% fetal bovine serum (FBS) (Gibco). HK-2, 786-O, 769-P, and SW839 were cultured in RPMI-1640 medium (Gibco) with 10% FBS. A-498 and ACHN were cultured in MEM medium (Gibco) with 10% FBS. Caki-1 and Caki-2 were cultured in McCoy’s 5a medium (Gibco) with 10% FBS. All cells were incubated in a humidified incubator (ThermoFisher) with 5% CO_2_.

### Chemicals

Camptothecin (MedChemExpress, HY-16560), olaparib (MedChemExpress, HY-10162), and anti-mouse PD-1 (Bio X Cell, BE0146) were used in this study.

### Construction of stable overexpression and knockdown cell lines

The plasmid transfection was performed with Lipofectamine 3000 Transfection Kit (Thermo Fisher Scientific) according to the manufacturer’s protocol. To generate indicated lentivirus, overexpression plasmid or shRNA targeting ACOX2 was transfected into HEK293T cells with pMD2.G and psPAX2 packaging plasmids. After 48 h, the cell supernatant containing lentivirus was harvested and passed through a 0.45-µm filter (Beyotime). To construct stable cell lines, the cells were infected with indicated lentivirus using polybrene (Sigma) and then screened with puromycin (Invivogen) for two weeks. The shRNA sequences targeting *ACOX2* were as follows: sequence #1, GCCATCAGTTATGCCTTCCAT; sequence #2, GGAAGGATGCCATCCTGTTAA.

### Cell Counting Kit-8 (CCK-8) assay

Cell proliferation was measured with CCK-8 assay kit (Beyotime). Cells were seeded into 96-well plates (2 × 10^3^ cells/well) with 100 µl complete culture medium. After incubation for 1, 2, 3, 4, and 5 days, 10 µl CCK-8 reagent was added to each well, followed by a 2-hour incubation period ensuring that light was avoided. Absorbance was measured at 450 nm with a Microplate Spectrophotometer (BioTek Instruments Inc.)

The IC_50_ values of olaparib in indicated 786-O, Caki-1, 769-P, and A-498 cells were assessed by CCK-8 assay. Cells were seeded into 96-well plates (5 × 10^3^ cells/well), and incubated in culture medium with gradient concentrations of olaparib for 72 h. Cell viability was measured with CCK-8 reagent as described above.

### Colony formation assay

Cells were seeded into 6-well plates (1 × 10^3^ cells/well) with 2 ml complete culture medium. After 14 days, cells were fixed with 4% paraformaldehyde (PFA) fix solution (Beyotime) and stained with crystal violet staining solution (Beyotime). Colonies consisting of more than 50 cells were counted.

### Transwell invasion assay

Cell invasive ability was evaluated with transwell chambers (Corning) placed in 24-well plates. A layer of Matrigel (Corning) was spread on the microporous membrane in the upper chamber. Cells were seeded into upper chamber with 200 µl FBS-free culture medium, while the lower chamber was filled with 800 µl complete culture medium. After 24 h incubation, the invasive cells were fixed with 4% PFA fix solution and stained with crystal violet staining solution.

### Wound healing assay

Cell migration ability was evaluated with wound healing assay. Cells were seeded in a six-well plate one day prior to the assay. When overgrown, wounds were created using 100-µl pipette tips. After 24 h, the wound gap area was photographed and measured.

### RNA extraction and real-time quantitative polymerase chain reaction (RT-qPCR)

Total RNA was extracted with TRIzol reagent (Thermo Fisher Scientific) according to the manufacturer’s protocol and quantified with a NanoDrop spectrophotometer (ThermoFisher). The cDNA was synthesized by the PrimeScript RT Reagent Kit (TaKaRa). The RT-qPCR was performed with SYBR Premix EX Taq (Takara) on the ABI 7900HT Real-Time PCR System (Applied Biosystems). GAPDH was used as the control.

Primer sequences used in this study were as follows (5’ to 3’): *ACOX2*-Forward, CGCCTGGGTTGGTTAGAAGAT; *ACOX2*-Reverse, CTGAGGGCTCTCACGAAGAC; *GAPDH*-Forward, ACAACTTTGGTATCGTGGAAGG; *GAPDH*-Reverse, GCCATCACGCCACAGTTTC.

### Enzyme-Linked Immunosorbent Assay (ELISA)

The protein abundance of IFN-α, IFN-β, CCL5, and CXCL10 in the cell supernatant was determined with the indicated ELISA kit (Shanghai Jianglai Biotechnology) according to the manufacturer’s instructions.

### Comet assay

The comet assay was performed with a comet assay kit (KeyGEN BioTECH) according to the manufacturer’s instructions. Briefly, cells were embedded on slides in 0.7% low-melting-point agarose and immersed in lysis buffer for 1 h at 4 °C. Cells were then incubated in alkaline electrophoresis buffer (1mmol/L EDTA, 300 mmol/L NaOH) for 30 min, and electrophoresed at 25 V for 30 min. Subsequently, cells were neutralized in 0.4 mM Tris-HCl (pH 7.5) buffer at 4 °C, followed by staining with propidium iodide (PI) for 10 min and photographed with confocal microscopy (Olympus). The Comet Assay Software Project (CASP) software was applied to analyze the percentage of tail DNA.

### DNA repair reporter assay

HEK293T-DR-GFP and HEK293T-EJ5-GFP reporter cell lines were constructed to evaluate the HRR and NHEJ efficiency. Briefly, the I-SceI plasmid was transfected into cells with Lipofectamine 3000 Transfection Kit to induce DNA damage. Cells were cultured for 48 h and harvested for further fluorescence activated cell sorting (FACS) analysis. The relative HRR and NHEJ efficiency were measured as percentage of GFP^+^ cells [56].

### Survival analysis

The Kaplan-Meier method was used to estimate survival curves for overall survival (OS), progression-free survival (PFS), and disease specific survival (DSS) of ccRCC patients with “survival” and “survminer” packages in R software as previously reported [57].

### Immunoblotting

Cells were lysed with RIPA Lysis Buffer (Beyotime) containing 1% protease and phosphatase inhibitors (Beyotime). Protein concentration was measured with BCA Protein Assay Kit (Beyotime). A total of 20 µg of proteins were separated by 5%, 10%, or 12.5% SDS-PAGE and transferred to polyvinylidene fluoride (PVDF) membrane (Millipore). After blocked with 5% skim milk in TBST buffer (Beyotime), the membranes were incubated with primary antibodies at 4 °C overnight and with secondary antibodies for 1 h at room temperature. The signal was visualized with LumiBest ECL substrate solution kit (ShareBio) and detected by the ChemiDocXRS system (Bio-Rad).

For immunoblotting analysis, primary antibodies included ACOX2 (ABclonal, #A12796, 1:500), GAPDH (Proteintech, #60004-1-Ig, 1:50000), MRE11 (Abcam, #ab214, 1:500), Flag (Cell Signaling Technology, #14793, 1:1000), Myc (Cell Signaling Technology, #ab32, 1:1000), RAD50 (Abcam, #ab124682, 1:1000), NBS1 (Abcam, #ab32074, 1:1000), γ-H2AX (Cell Signaling Technology, #9718, 1:1000), H2AX (Cell Signaling Technology, #7631, 1:1000), CtIP (Cell Signaling Technology, #9201, 1:1000), RAD51 (Abcam, #ab88572, 1:1000), RPA32 (Abcam, #ab76420, 1:1000), 53BP1 (Proteintech, #20002-1-AP, 1:500), RIF1 (Proteintech, #30119-1-AP, 1:1000), cGAS (Cell Signaling Technology, #79978, 1:1000), STING (Cell Signaling Technology, #13647, 1:1000), p-STING (Cell Signaling Technology, #50907, 1:1000), TBK1 (Abcam, #ab40676, 1:1000), p-TBK1 (ABclonal, #AP0847, 1:500), IRF3 (Proteintech, #11312-1-AP, 1:500), and p-IRF3(ABclonal, #AP0623, 1:500). Secondary antibodies were anti-mouse IgG(H + L) (Proteintech, # SA00001-1, 1:5000) and anti-rabbit IgG(H + L) (Proteintech, # SA00001-2, 1:5000).

### Co-immunoprecipitation (Co-IP)

The cell supernatant lysates were incubated with 2 µg of antibody and 20 µl of protein A or protein G sepharose beads overnight at 4 °C, and further experiments were performed with an immunoprecipitation kit (Beyotime) according to the manufacturer’s protocol. Immunoblotting was performed following standard procedures.

### Molecular docking analysis

Rigid protein–protein docking between ACOX2 and MRE11 was performed to investigate the interacting amino acid residue sites with GRAMM Docking web server (http://gramm.compbio.ku.edu/) [58]. The protein structural domains of ACOX2 and MRE11 were obtained from the AlphaFold Protein Structure Database (https://alphafold.ebi.ac.uk/). The PDBePISA database (https://www.ebi.ac.uk/pdbe/pisa/) and Pymol software (Version 3.0) were used to explore protein-protein interactions and further visual analysis [59].

### GST pull-down

Whole-cell lysates containing His-MRE11 protein were prepared in HEK293T cells. GST and GST-fusion ACOX2 proteins were prepared in bacteria, and were purified by binding to glutathione-sepharose resins for 3 h at 4 °C. Whole-cell lysates containing His-MRE11 protein were incubated with the indicated purified proteins overnight at 4 °C. After being eluted with elution buffer five times, the bound proteins were separated by SDS–PAGE and analyzed by immunoblotting.

### Immunocytochemistry (ICC) staining

Cells were cultured on 35 mm confocal dishes (Biosharp) and treated with CPT for the indicated concentrations and time. The prepared cells were fixed with 4% PFA for 30 min, permeabilized in 1% Triton X-100 solution (Beyotime) for 10 min, and blocked with 5% BSA (ShareBio) for 1 h at room temperature. Then, cells were incubated with primary antibody overnight at 4 °C and with secondary antibody for 1 h at room temperature. Antifade mounting medium with DAPI was added to the dishes for 30 min. The samples were visualized with confocal microscopy (Olympus).

For ICC analysis, primary antibodies included ACOX2 (Proteintech, # 17571-1-AP, 1:50), MRE11 (Abcam, #ab214, 1:100), γ-H2AX (Cell Signaling Technology, #9718, 1:200), CtIP (Cell Signaling Technology, #9201, 1:100), RAD51 (Abcam, #ab88572, 1:100), RPA32 (Abcam, #ab76420, 1:50), 53BP1 (Proteintech, #20002-1-AP, 1:100), and RIF1 (Proteintech, #30119-1-AP, 1:200). Secondary antibodies were anti-mouse IgG (H + L) Alexa Fluor 488 Conjugate (Cell Signaling Technology, #4408, 1:2000), anti-mouse IgG (H + L) Alexa Fluor 555 Conjugate (Cell Signaling Technology, #4409, 1:2000), anti-rabbit IgG (H + L) Alexa Fluor^®^ 488 Conjugate (Cell Signaling Technology, #4412, 1:2000), and anti-rabbit IgG (H + L) Alexa Fluor^®^ 555 Conjugate (Cell Signaling Technology, #4413, 1:2000).

### Immunohistochemistry (IHC) and hematoxylin-eosin (H&E) staining

IHC staining involved the following steps, including dewaxing, antigen repair, blocking endogenous peroxidase, serum closure, primary antibody incubation, secondary antibody incubation, diaminobenzidine solution (DAB) color development, restaining nuclei, dewatering, sealing, and microscope inspection. The IHC score was assessed by two independent pathologists under the same conditions. The staining percentage and intensity were graded as follows: 0, 1, 2, 3, or 4; and 0, 1, 2, or 3, respectively. The final IHC scores were calculated by multiplying the percentage and intensity scores. H&E staining was performed as follows: dewaxing, hematoxylin staining, eosin staining, dehydration, sealing, and microscope inspection. IHC and H&E slides were visualized in SlideViewer software. The detailed methodology of IHC and H&E staining was described as previously reported [35].

For IHC analysis, primary antibodies included ACOX2 (Proteintech, # 17571-1-AP, 1:100), γ-H2AX (Cell Signaling Technology, #9718, 1:500), CD8 (Proteintech, #66868-1-Ig, 1:10000), p-STING (Cell Signaling Technology, #50907, 1:100), and p-IRF3(ABclonal, #AP0623, 1:100). Secondary antibodies were HRP conjugated Goat Anti-Mouse IgG (H + L) (Servicebio, 1:10000) and HRP conjugated Goat Anti-Rabbit IgG (H + L) (Servicebio, 1:10000).

### Multiplex immunohistochemistry (mIHC) staining

The mIHC staining was performed with Opal polaris 7 color automation IHC detection kit (AccuraMed) following the manufacturer’s instructions. Briefly, FFPE tissue samples of ccRCC patients were cut into sections of 4 μm thickness with sequential staining cycles. The paraffin-embedded sections were deparaffinized and rehydrated. Antigen retrieval was performed in EDTA buffer with microwave heating. After blocked, the sections were incubated with primary antibodies at 4 °C overnight and further incubated with HRP-conjugated secondary antibodies at room temperature for 1 h. Subsequently, tyramide signal amplification (TSA) was applied to the sections. After every staining cycle, antigen retrieval was repeated to remove the antibody-TSA complex. After all the antigens were labelled, the TSA-stained slides were stained with DAPI.

### Establishment of patient-derived organoid (PDO) model

The fresh ccRCC tissues were placed in the cold PBS with normocin (InvivoGen) and gentamicin/amphotericin B (GIBCO) for tumor cell isolation and culture. In the lab, tissues were washed in the cold PBS with penicillin/streptomycin (Gibco) for 5 × 5 min, and minced into tiny fragments in the sterile dish on ice. The enzymatic digestion was performed in mixed digestion medium for 2 h at 37 °C. Then, the digested tissues were centrifuged for 5 min at 500 g, and seeded in a well of pre-warmed 24-well culture plate precoated Matrigel (D1 Medical Technology). The PDO was cultured with 500 ul ccRCC PDO culture medium (D1 Medical Technology) in 37 °C incubator with 5% CO_2_. The culture medium was replaced every three days. The PDO morphology was observed and photographed at indicated time. The PDO viability was evaluated by CellTiter-Glo 3D Cell viability assay kit (Promega) according to manufacturer’s instruction.

For HE, IHC, and mIHC stainings of PDO, organoids were fixed in pre-warmed 4% PFA at 37 °C for 30 min after removing the culture medium. Then organoids in polymerized Matrigel were transferred into the round hole of a cassette and subjected to staining procedures. The primary antibodies used in PDO staining included PAX8 (GeneTech, GT210204), CA-9 (GeneTech, GT224004), and E-Cadherin (GeneTech, GT234807).

### Establishment of mouse models

For cell-derived xenograft (CDX) model, 1 × 10^6^ indicated 786-O or Caki-1 cells were transplanted into 6-week-old BALB/c nude mice. The mice were euthanized by CO_2_ suffocation on day 24, and xenograft tumors were dissected for further analysis. For patient-derived xenograft (PDX), immunodeficient NPSG mice (Phenotek) were used. Fresh human ccRCC tumor fragments were implanted into the flanks of mice. When tumors reached 2 cm in diameter, they were collected and reimplanted into new mice. Starting from day 10, mice were intraperitoneally injected with olaparib (MedChemExpress) every 2 days with a dose of 25 mg/kg. The mice were euthanized by CO_2_ suffocation on day 46, and xenograft tumors were dissected for further analysis. To evaluate the treatment response to olaparib and anti-PD-1, 1 × 10^6^ indicated Renca cells were transplanted into 6-week-old BALB/c immunocompetent mice. Starting from day 6, mice were intraperitoneally injected with olaparib every 2 days with a dose of 25 mg/kg or anti-PD-1 (Selleck) every 2 days with a dose of 200 µg. The mice were euthanized by CO_2_ suffocation on day 46, and tumors were dissected for further analysis. All animal experiments were performed according to protocols approved by the Institutional Animal Care and Use Committee (IACUC) of Fudan University Shanghai Cancer Center (FUSCC). The tumor volume was calculated using the following formula: tumor volume = 0.5 × length × width × width. The tumor diameter did not exceed the IACUC-approved maximum tumor diameter of 2 cm.

### Flow cytometry analysis

The fresh dissected mouse tumors were mechanically minced and digested with a Tumor Dissociation Kit (Miltenyi) according to the manufacturer’s protocol. The dissociated cell suspensions were passed through a 70-µm cell strainer (Beyotime) and then lysed with red blood cell lysis buffer (Abcam). Then, the cell suspensions were washed in flow cytometry staining buffer and incubated with the indicated antibodies at 4 °C for 1 h. The antibodies applied for flow cytometry analysis included anti-CD45 (BioLegend, #103113), anti-CD3 antibody (BioLegend, #100306), anti-CD8 antibody (BioLegend, #100766), anti-Interferon γ (BioLegend, #505807), anti-Granzyme B (BioLegend, #372211), anti-PD-1 antibody (BioLegend, #135220), anti-Tim-3 (BioLegend, #119716).

The co-cultured T cells were collected for subsequent suspension with cell staining buffer (Biolegend, 420201), fixation with fixation Buffer (Biolegend, 420801), permeabilization with intracellular staining permeabilization wash buffer (Biolegend, 421002). Then, the cells were incubated with the indicated antibodies at 4 °C for 1 h. The antibodies applied for flow cytometry analysis included: anti-CD3 antibody (BioLegend, # 317317), anti-CD8 antibody (BioLegend, #301032), anti-Interferon γ (BioLegend, #502527), and anti-Granzyme B (BioLegend, #396406).

The cell apoptosis assay was performed with Annexin V-PE/7-AAD Apoptosis Kit (Multi Sciences) following the manufacturer’s instructions. The flow cytometric data were analyzed by CytExpert software (Version 2.4).

### Protein Extraction and Tryptic Digestion for LC-MS/MS

Samples were lysed in 100 µL TCEP buffer (2% deoxycholic acid sodium salt, 40 mM 2-chloroacetamide, 100 mM Tris-HCl, 10 mM Tris (2-chloroethyl) phosphate, 1 mM PFSM, pH 8.5) supplemented with protease inhibitors at 99 ℃ for 30 min. After cooling to room temperature, trypsin (Promega, Madison, WI, USA, #V5280) was added and digested for 18 h at 37 °C. 10% formic acid was added and vortexed for 3 min, followed by sedimentation for 5 min (12,000 g). Next, a new 1.5-mL tube with extraction buffer (0.1% formic acid in 50% acetonitrile) was used to extract the supernatant (vortex for 3 min, followed by 12,000 g of sedimentation for 5 min). Collected supernatant was transferred into a new tube for drying using a SpeedVac. After drying, 100 µL 0.1% FA was needed for dissolving the peptides and vortex for 3 min, and then sedimentation for 3 min (12,000 g). The supernatant was picked into a new tube and then desalinated. Before desalination, the activation of pillars with 2 slides of 3 M C8 disk was required, and the liquid was as follows: 90 µL 100% ACN twice, 90 µL 50% and 80% ACN once in turn, and then 90 µL 50% ACN once. After pillar balance with 90 µL 0.1% FA twice, the supernatant of the tubes was loading into the pillar twice, and decontamination with 90 µL 0.1% FA twice. Lastly, 90 µL elution buffer (0.1% FA in 50% ACN) was added into the pillar for elution twice and only the eluent was collected for MS. And then the collection liquid was put in a 60 °C vacuum drier for drying (∼1.5 h). The subsequently detailed methodology of LC-MS/MS was described as previously reported [60, 61].

### LC-MS/MS analysis

The peptides were analyzed on FAIMS interfaced Orbitrap Fusion Lumos Tribrid Mass Spectrometer (Thermo Fisher Scientific, Rockford, IL, USA) equipped with an Easy nLC-1000 (Thermo Fisher Scientific, Rockford, IL, USA) and a Nanoflex source (Thermo Fisher Scientific, Rockford, IL, USA). Dried peptide samples were re-dissolved in buffer A (0.1% FA in water) were loaded to a 2 cm self-packed trap column using buffer A and separated on a 150 μm inner diameter column with a length of 30 cm over a 150 min gradient (buffer A: 0.1% FA in water; buffer B: 0.1% FA in 80% ACN) at a constant flow rate of 600 nL/min (0–150 min, 0 min, 4% B; 0–10 min, 4−15% B; 10–125 min, 15–30% B; 125–140 min, 30–50% B; 140–141 min, 50–100% B; 141–150 min, 100% B). The eluted peptides were ionized and detected. Compensation Voltages (CV) among − 30 V, −60 V, and − 120 V were interrogated to find precursor rich CVs. Mass spectra were acquired over the scan range of m/z 350–1500 at a resolution of 120,000 (AUG target value of 5E5 and max injection time 50 ms). For the MS2 scan, the higher-energy collision dissociation fragmentation was performed at a normalized collision energy of 30%. The MS2 AGC target was set to 1e4 with a maximum injection time of 10 ms, peptide mode was selected for monoisotopic precursor scan, and charge state screening was enabled to reject unassigned 1+, 7+, 8+, and > 8 + ions with a dynamic exclusion time of 45 s to discriminate against previously analyzed ions between ± 10 ppm.

### Peptide identification and protein quantification

In our study, all MS raw files were processed at firmiana platform (a one-stop proteomic cloud platform: http://www.firmiana.org) [62]. Briefly, all MS raw files were searched against the National Center for Biotechnology Information (NCBI) human RefSeq protein database (updated on 04-07-2013, 32,015 entries) in Mascot search engine (version 2.3, Matrix Science Inc). Trypsin was used as the proteolytic enzyme allowing up to two missed cleavages. Carbamidomethyl (C) was considered as a fixed modification. For the proteome profiling data, variable modifications were oxidation (M) and acetylation (Protein N-term). All the identified peptides were quantified at firmiana platform with peaks area derived from their MS1 intensity. The mass tolerances were 10 ppm for precursor and 10 ppm for the product, which has been applied in previously published studies [63]. Precursor ion score charges were limited to + 2, +3, and + 4. The FDRs of the peptide-spectrum matches (PSMs) and proteins were set at a maximum 1%. The same cutoff strategies of FDR have been widely used in recently published research [64, 65]. Label-free protein quantifications were calculated in our cohortusing the so-called intensity-based absolute quantification (iBAQ) algorithm, which divided the protein abundance (derived from identified peptides’ intensities) by the number of theoretically observable peptides.

### Data normalization

Identified proteins were normalized using the fraction of total (FOT) method, where a relative quantification value is defined as a protein’s iBAQ divided by the total iBAQ of all identified proteins in one experiment [66]. Then, the FOT was further multiplied by 1e6 for presentation ease. Finally, the FOT values were calculated for all samples and used in all subsequent quantitative analyses to correct sample loading differences.

### Differentially expressed proteins (DEPs) analysis

A total of 8,704 proteins identified in 10 samples were used for differential expression analysis. The protein expression matrix was used to identify DEPs compared the ACOX2-OE group to Vector group with Contrasts function implemented with the limma R package. After adjusting the *p* value with the Benjamini-Hochberg (BH) method, 306 DEPs (261 upregulation and 45 downregulation) were identified in ACOX2-OE group vs. Vector group (Wilcoxon rank-sum test, BH *p* < 0.05, ACOX2-OE/Vector > 2 or < 0.5).

### Pathway enrichment analysis

To investigate the dominant signaling pathways in ACOX2-OE group, we used gene sets of upregulated DEPs (*n* = 261) in ACOX2-OE group vs. Vector group, which were enriched by KEGG (RRID: SCR_012773)/GO (RRID: SCR_002811) database and ConsensusPathwayDB (http://cpdb.molgen.mpg.de/, RRID: SCR_002231). We then annotated the signaling pathways (BH *p* < 0.05) and manually checked the pathway associated proteins (Wilcoxon rank-sum test, BH *p* < 0.05).

### Principal component analysis (PCA)

We performed PCA on a total of 8,704 proteins identified in 10 samples to illustrate the global proteomic difference between Vector group and ACOX2-OE group. The PCA function under the scikit-learn R package was implemented for unsupervised clustering analysis with the parameter “n_components = 2” on the expression matrix of global proteomic data. A colored ellipse represented the 95% confidence coverage for each group, calculated based on the mean and covariance of points in each specific group.

### ACOX2-interacting proteins detected in 786-O cells using LC–MS/MS

786-O cells were transfected with Flag-ACOX2 plasmid for 48 h, and then lysed on ice in 0.1% NP40 buffer (Beyotime, P0013F) for 30 min. Insoluble cell debris was removed by centrifugation, and cell lysates were incubated with anti-Flag beads (Sigma, M8823) at 4 ℃ overnight. After IP assay, the sample was loaded on 10% SDS-PAGE gel and then visualized with fast silver stain kit (Beyotime, P0017S) according to the manufacturer’s instructions. The sample was subsequently analyzed for LC-MS/MS to identify the ACOX2-interacting proteins.

### Statistical analysis

All data were presented as mean ± SD unless otherwise specified. The statistical tests applied were indicated in the figure legends. Statistical analysis was performed with GraphPad Prism software (Version 9.0) and R software (Version 4.2.3). Difference was significant when *p* value was < 0.05.

## Results

### ACOX2 downregulation is observed in ccRCC and correlated with a worse prognosis

**Fig. 1:**
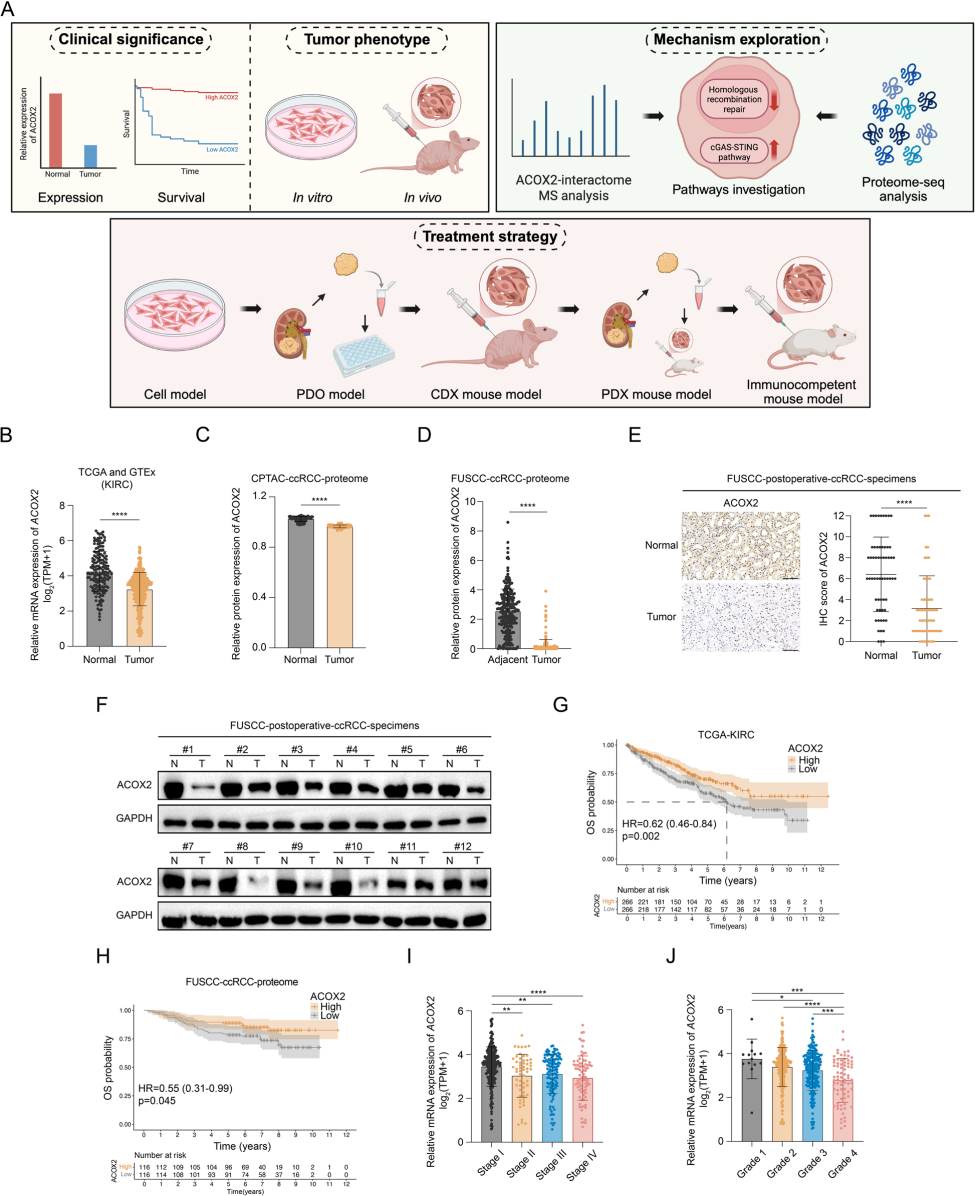
ACOX2 downregulation in ccRCC is associated with a poorer prognosis. **A** The research flowchart of the research. **B** Relative mRNA expression of *ACOX2* in tumor and normal tissues of KIRC from TCGA and GTEx databases. **C** Relative protein expression of ACOX2 in ccRCC and normal tissues from CPTAC database. **D** Relative protein expression of ACOX2 in ccRCC and paired adjacent tissues from FUSCC-ccRCC cohort. **E** Representative immunohistochemistry (IHC) micrographs and quantification data of ACOX2 in ccRCC and paired normal tissues from 60 postoperative specimens. Scale bar: 100 μm. **F** Immunoblotting of ACOX2 in ccRCC and paired normal tissues from 12 postoperative specimens. **G** Kaplan–Meier survival curves for overall survival (OS) of ccRCC patients with low or high expression of *ACOX2* from TCGA-KIRC cohort. **H** Kaplan–Meier survival curve for OS of ccRCC patients with low or high expression of ACOX2 from FUSCC-ccRCC cohort. **I**,** J** Correlation between *ACOX2* mRNA expression and clinical stage (**I**) or grade (**J**) in ccRCC patients from TCGA-KIRC cohort. Statistical significance was determined by two-tailed unpaired t-test (**B, C**, **I**, **J** ), two-tailed paired t-test (D, E), and the two-sided log-rank (Mantel–Cox) test (**G**, **H**). * *P* < 0.05, **P < 0.01, *** *P* < 0.001, **** *P* < 0.0001, and ns *P* ≥ 0.05. Experiments were independently repeated three times with similar results; data of one representative experiment are shown (**E**, **F**)

The research flowchart is presented in Fig. 1A. To investigate the role of ACOX2 in ccRCC, we first assessed its expression and prognostic significance across multi-omics datasets. Integration of RNA-seq data from TCGA and GTEx databases revealed significant downregulation of *ACOX2* mRNA in ccRCC (KIRC) compared to normal kidney tissues (Fig. 1B). This downregulation was also evident in papillary RCC (KIRP, pRCC) and chromophobe RCC (KICH, chRCC) (Fig. S1A-B). Similarly, protein expression analysis within the CPTAC-ccRCC cohort demonstrated reduced ACOX2 level in ccRCC tissues (Fig. 1C). Further corroboration came from our previously reported FUSCC-ccRCC cohort (including 232 paired ccRCC and adjacent normal specimens), which showed a marked decrease in ACOX2 protein expression (Fig. 1D) [67]. Validation using IHC on 60 paired surgical specimens confirmed significant ACOX2 reduction in ccRCC tissues, and IB of 12 paired samples further substantiated this downregulation (Fig. 1E-F). Moreover, higher *ACOX2* mRNA expression was associated with better clinical outcomes, including OS, PFS, and DSS (Fig. 1G and S1C-D). This association with superior prognosis was also observed at the protein level in the FUSCC-ccRCC cohort (Fig. 1H). Additionally, higher ACOX2 mRNA expression correlated strongly with excellent tumor-node-metastasis (TNM) stage and pathologic grade (Fig. 1I-J). Collectively, these findings, validated across multi-omics cohorts, establish the clinical significance of ACOX2 expression and its prognostic value in ccRCC.

### ACOX2 suppresses the tumorigenic properties of ccRCC in vitro and in vivo

**Fig. 2:**
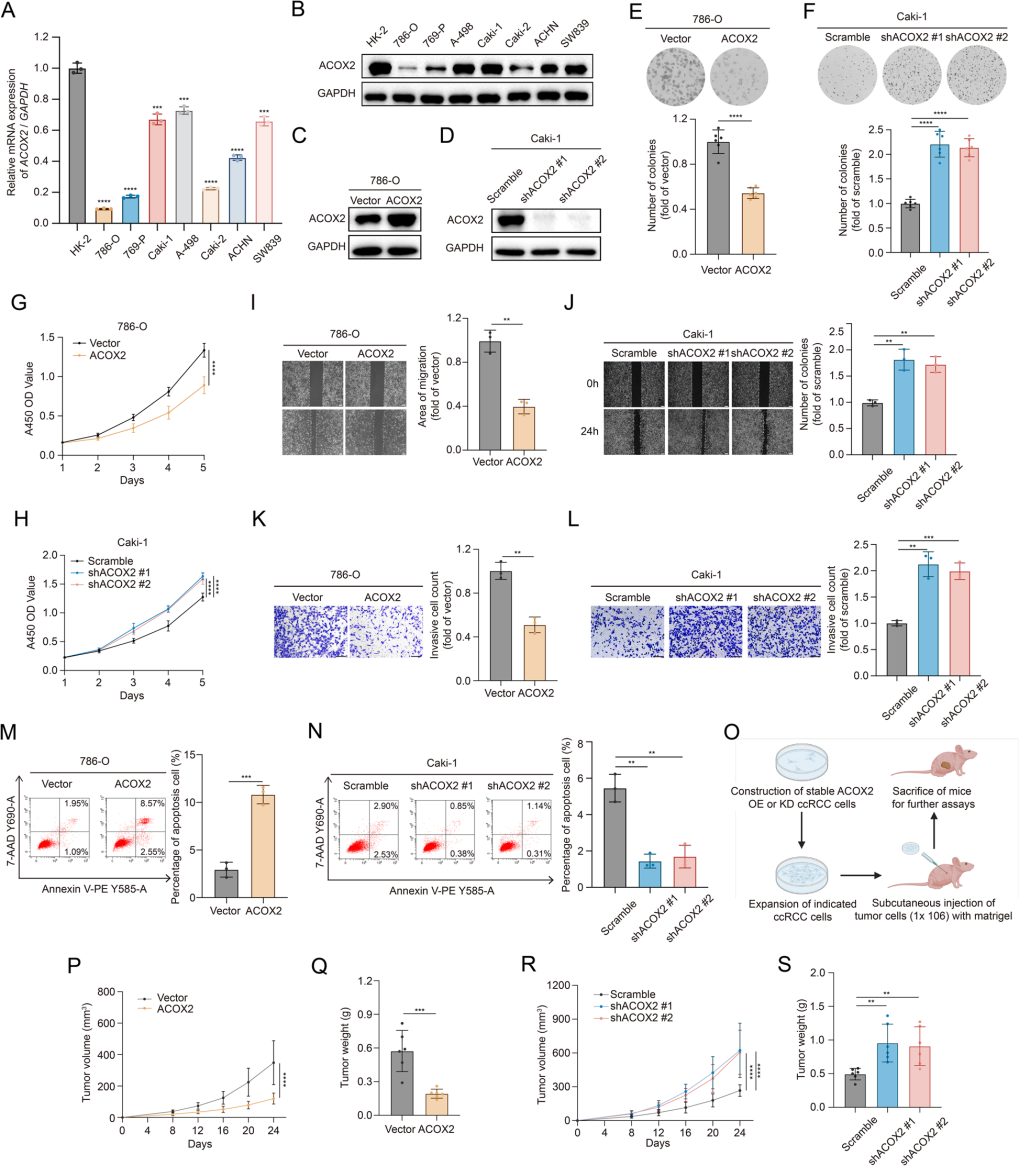
ACOX2 inhibits the biological characteristics of ccRCC.**A**,** B** Quantitative polymerase chain reaction (qPCR) (**A**) and immunoblotting (**B**) of ACOX2 in HK-2 and seven RCC cell lines. **C**,** D** Immunoblotting of ACOX2 in the indicated 786-O (**C**) and Caki-1 (**D**) cells. **E**,** F** Colony formation assay of the indicated 786-O (**E**) and Caki-1 (**F**) cells. **G**,** H** Growth curves of the indicated 786-O (**G**) and Caki-1 (**H**) cells using Cell Counting Kit-8 (CCK-8). **I**,** J** Wound healing assay of the indicated 786-O (I) and Caki-1 (**J**) cells. Scale bar: 200 μm. **K**,** L** Transwell invasive assay of the indicated 786-O (**K**) and Caki-1 (**L**) cells. Scale bar: 200 μm. **M**,** N** Percentage of apoptosis cell of the indicated 786-O (**M**) and Caki-1 (**N**) cells with flow cytometry analysis. **O** Schematic illustration for the generation of ccRCC cell-derived xenograft (CDX) model. **P**,** Q** The growth curves (**P**) and tumor weight (**Q**) of the indicated 786-O CDX. **R**,** S** The growth curves (**R**) and tumor weight (**S**) of the indicated Caki-1 CDX. Statistical significance was determined by two-tailed unpaired t-test (**A**, **E**-**N**, **Q**, **S**) and two-way analysis of variance (ANOVA) (**P**, **R**). * *P* < 0.05, **P < 0.01, *** *P* < 0.001, **** *P* < 0.0001, and ns *P* ≥ 0.05. Experiments were independently repeated three times with similar results; data of one representative experiment are shown (**B**-**F**, **I**-**N**)

Given the potential tumor-suppressive role of ACOX2 in ccRCC, we further explored its exact function and mechanism. Compared to human renal cortex proximal tubule epithelial cell (HK-2), ACOX2 expression was notably downregulated in seven RCC cell lines, particularly in 786-O and 769-P (Fig. 2A-B). Next, we established stable ACOX2-overexpression (ACOX2-OE) (786-O) and ACOX2-knockdown (ACOX2-KD) (Caki-1) cell lines (Fig. 2C-D). Colony formation and CCK-8 assays demonstrated that ACOX2 inhibited ccRCC proliferation in vitro (Fig. 2E-H). Wound healing assay indicated that ACOX2 upregulation decreased ccRCC migratory potential (Fig. 2I-J). Transwell assay showed that ACOX2 inhibited the invasive ability of ccRCC cells (Fig. 2K-L). Flow cytometry analysis revealed that ACOX2 increased the proportion of apoptotic cells (Fig. 2M-N). These in vitro results were consistent across two additional ccRCC cell lines (769-P and A-498) (Fig. S2A-L). To further validate our findings in vivo, we constructed CDX models with 786-O and Caki-1 cells in BALB/c nude mice (Fig. 2O). Consistently, ACOX2 significantly inhibites ccRCC tumor growth (Fig. 2P-S and S2M-N).

### ACOX2 interacts with MRE11 and destabilizes the MRE11-RAD50-NBS1 complex

**Fig. 3:**
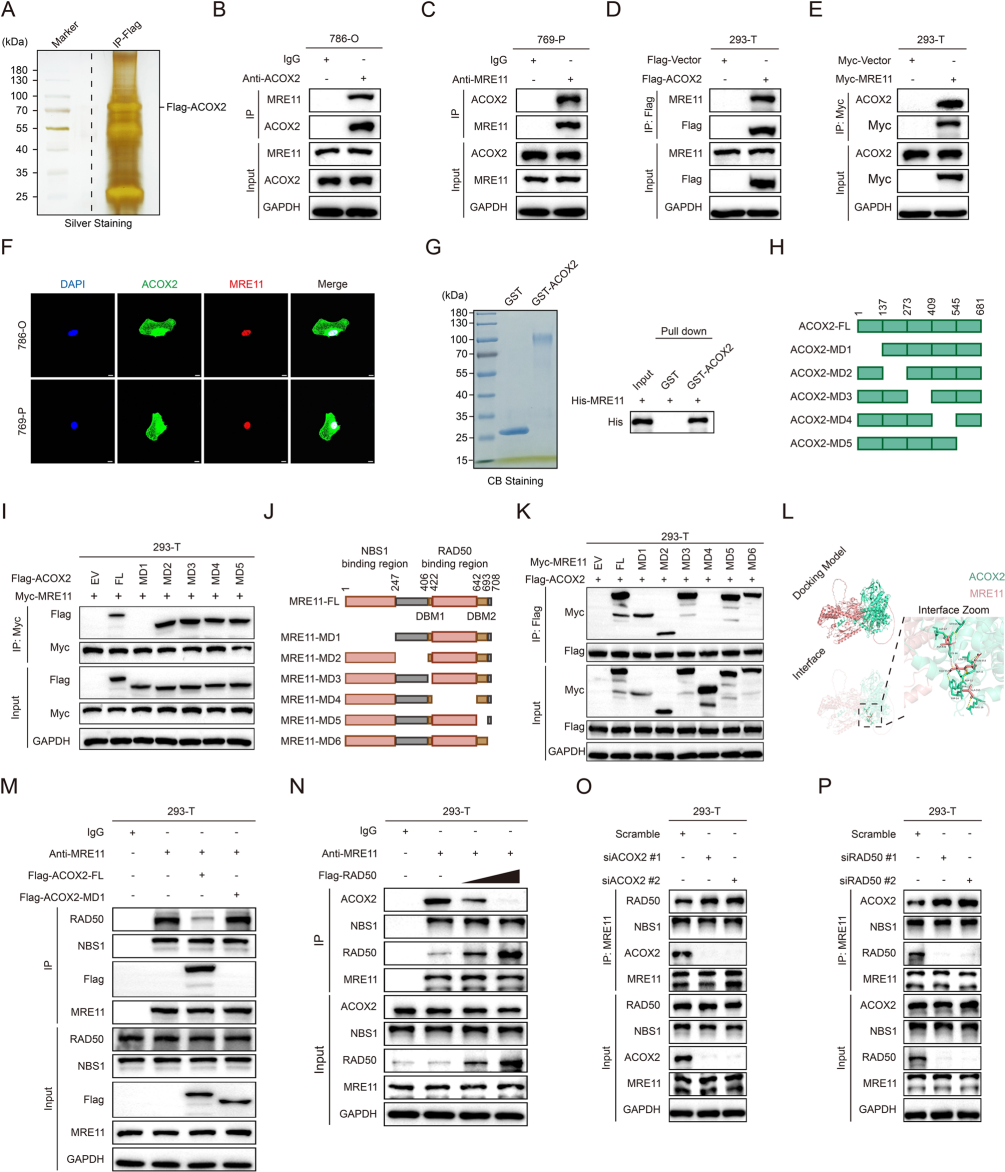
ACOX2 interacts with MRE11 and disrupts the stability of the MRE11-RAD50-NBS1 complex.**A** The proteins were separated by SDS-PAGE and visualized by silver staining for further LC-MS/MS analysis. The sample was collected by immunoprecipitation (IP) assay using anti-Flag beads from 786-O cells transfected with Flag-ACOX2 plasmid. **B**,** C** Co-IP between endogenous ACOX2 and MRE11 in 786-O (B) and 769-P (**C**) cells. **D** Co-IP between MRE11 and exogenous ACOX2 in HEK293T cell. **E** Co-IP between ACOX2 and exogenous MRE11 in HEK293T cell. **F** Intracellular localization of endogenous ACOX2 and MRE11 in 786-O and 769-P cells. Scale bar: 10 μm. **G** GST pull-down assay of his-MRE11 combined with GST or GST-ACOX2. **H** Schematic illustration of ACOX2 truncated mutants. **I** Co-IP between indicated ACOX2 truncated mutants with MRE11. **J** Schematic illustration of MRE11 truncated mutants. **K** Co-IP between indicated MRE11 truncated mutants with ACOX2. **L** Diagram of the docking model and interacting amino acid between ACOX2 and MRE11. (ACOX2, green; MRE11, pink; hydrogen bond interaction, yellow dotted line). **M** Co-IP between MRE11 and RAD50, NBS1, or Flag-ACOX2, with or without Flag-ACOX2-FL or Flag-ACOX2-MD1 mutant overexpression. **N** Co-IP between MRE11 and ACOX2, NBS1, or RAD50, with or without gradient RAD50 overexpression. **O** Co-IP between MRE11 and RAD50, NBS1, or ACOX2, with or without ACOX2 knockdown. **P** Co-IP between MRE11 and RAD50, NBS1, or ACOX2, with or without RAD50 knockdown. Experiments were independently repeated three times with similar results; data of one representative experiment are shown (**A**-**G**, **I**, **K**, **M**-**P**)

To elucidate the mechanism underlying ACOX2’s regulation of ccRCC, we performed IP assay using anti-Flag beads to capture ACOX2-interacting proteins from lysates of 786-O cells overexpressing Flag-ACOX2. The silver staining assay confirmed significant enrichment of Flag-ACOX2 (Fig. 3A). Subsequent LC-MS/MS analysis identified MRE11 as a high-confidence interactor (Supplementary Table 3A). MRE11 is the core component of the MRN complex, responsible for initiating HRR and orchestrating DNA repair [21, 22]. Given our prior findings that ACOX2 regulated HCC through DDR-related pathway, we hypothesized that ACOX2 may interact with MRE11 to modulate ccRCC [47]. The interaction between ACOX2 and MRE11 was validated in ccRCC cells (786-O and 769-P) by co-IP (Fig. 3B-C). Exogenous expression also confirmed a strong ACOX2-MRE11 interaction in HEK293T cells (Fig. 3D-E). ICC assay showed ACOX2 and MRE11 colocalized in the nucleus (Fig. 3F). The GST pull-down assay further confirmed the direct interaction between ACOX2 and MRE11 (Fig. 3G). To pinpoint the exact structural regions responsible for the ACOX2–MRE11 interaction, we generated serial truncated mutants of ACOX2 and MRE11 (Fig. 3H and J). Co-IP assays showed deletion of the MD1 domain in ACOX2 or the RAD50-binding region (MD4) in MRE11 abolished the interaction (Fig. 3I and K). Protein-protein docking analysis predicted a stable interaction interface within these domains **(**Fig. 3L). As MRE11 typically functions within the MRN complex to regulate HRR, we examined ACOX2’s effect on the complex stability. Co-IP confirmed ACOX2 bound to MRE11 but not RAD50 or NBS1 (Fig. S3A). ACOX2 did not alter the complex component expression (Fig. S3B). Crucially, ACOX2 inhibited MRE11-RAD50 binding, and reciprocally, RAD50 hindered MRE11-ACOX2 binding (Fig. 3M-N). Knockdown of either ACOX2 or RAD50 enhanced the binding of MRE11 to the other partner (Fig. 3O-P). Together, these results indicate ACOX2 destabilizes the MRN complex by competing with RAD50 for MRE11 binding.

### ACOX2 inhibits HRR and aggravates DSBs by disrupting the MRN complex assembly

**Fig. 4:**
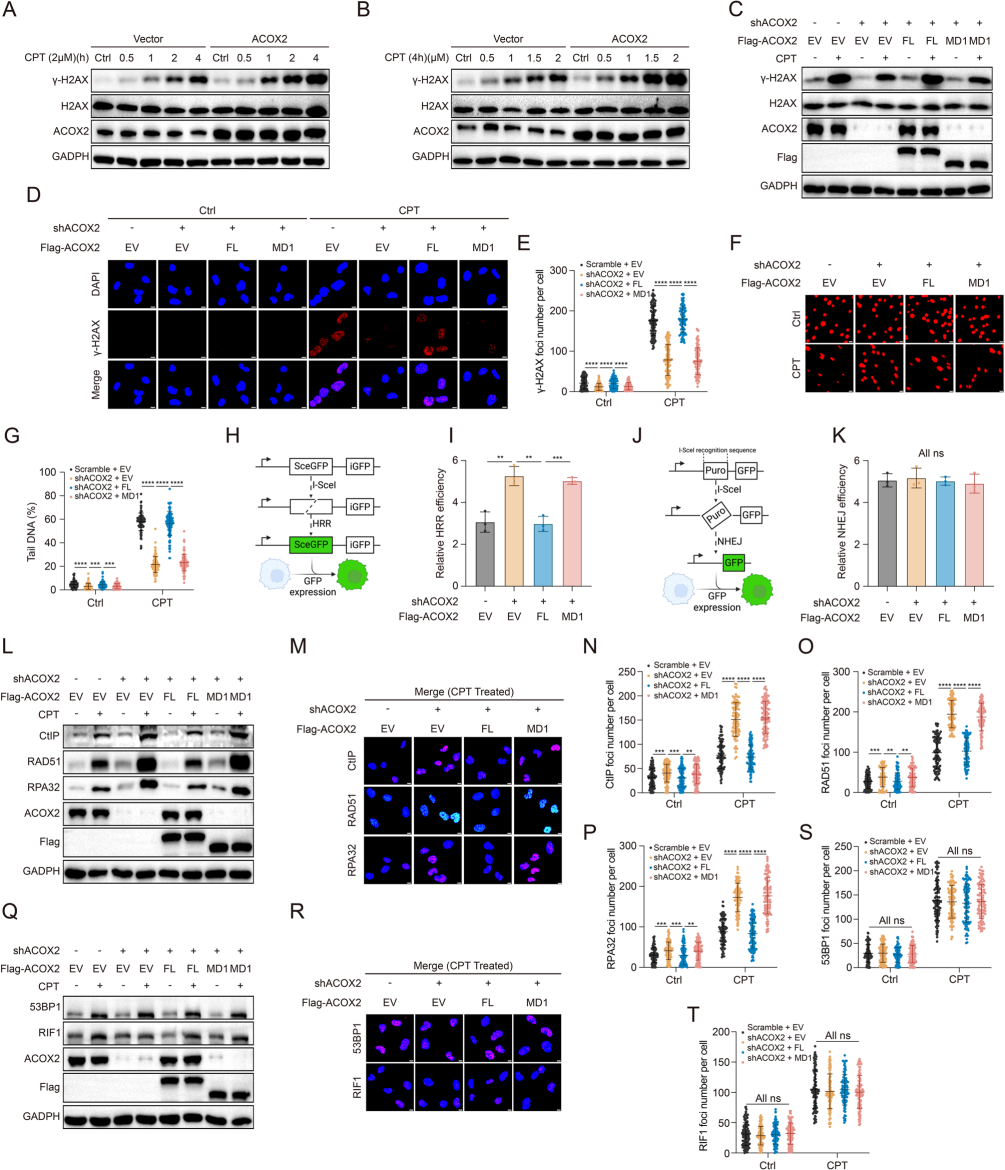
ACOX2 inhibits homologous recombination repair and increases double-strand breaks in ccRCC cell.**A** 786-O cells were treated with or without camptothecin (CPT) (2 μM) at the specific time, and cell lysates were immunoblotted with the indicated antibodies. **B** 786-O cells were treated with or without CPT (4 h) at the specific concentrations, and cell lysates were immunoblotted with the indicated antibodies. **C** 786-O cells were treated with or without CPT (2 μM, 4 h), and cell lysates were immunoblotted with the indicated antibodies. **D, E** Representative micrographs (**D**) and quantification data (**E**) for γ-H2AX foci formation in the indicated 786-O cells treated with or without CPT (2 μM, 4 h). Scale bar: 10 μm. **F, G** Representative micrographs (**F**) and quantification data (**G**) of comet assay in the indicated 786-O cells treated with or without CPT (2 μM, 4 h). Scale bar: 20 μm. **H** Schematic illustration of the DR-GFP reporter system. **I** Relative homologous recombination repair (HRR) efficiency in indicated HEK293T cells. **J** Schematic illustration of the EJ5-GFP reporter system. **K** Relative non-homologous end joining (NHEJ) efficiency in indicated HEK293T cells. **L** 786-O cells were treated with or without CPT (2 μM, 4 h), and cell lysates were immunoblotted with the indicated antibodies. **M-P** Representative micrographs (**M**) and quantification data (**N**-**P**) for CtIP, RAD51, and RPA32 foci formation in the indicated 786-O cells treated with or without CPT (2 μM, 4 h). Scale bar: 10 μm. **Q** 786-O cells were treated with or without CPT (2 μM, 4 h), and cell lysates were immunoblotted with the indicated antibodies. **R-T** Representative micrographs (**R**) and quantification data (**S**, **T**) for 53BP1 and RIF1 foci formation in the indicated 786-O cells treated with or without CPT (2 μM, 4 h). Scale bar: 10 μm. Statistical significance was determined by two-tailed unpaired t-test (**E**, **G**, **I**, **K**, **N**-**P**, **S**, **T**). * P < 0.05, **P< 0.01, *** P < 0.001, **** P < 0.0001, and ns *P* ≥ 0.05. Experiments were independently repeated three times with similar results; data of one representative experiment are shown (**A**-**D**, **F**, **L**, **M**, **Q**, **R**)

Given the MRN complex’s essential roles in DNA damage surveillance and HRR coordination, we investigated ACOX2’s potential involvement in DDR. Upon camptothecin (CPT)-induced DNA damage, ACOX2-OE significantly increased expression of γ-H2AX, a widely used DSBs marker, in ccRCC cells (Fig. 4A-B and Fig. S4A-B). Conversely, ACOX2-KD decreased the expression and foci formation of γ-H2AX, while re-expressing the MRE11-binding-deficient ACOX2-MD1 mutant did not reverse the effect, indicating that ACOX2-induced DSBs accumulation depends on MRE11 binding (Fig. 4C-E and Fig. S4C-E). Comet assay confirmed that ACOX2 enhanced DSBs accumulation (Fig. 4F-G and Fig. S4F-G).

Furthermore, we applied the DR-GFP and EJ5-GFP plasmid reporter systems to evaluate the role of ACOX2 in two main DSBs repair pathways, HRR and NHEJ. The results suggested that ACOX2 significantly impaired HRR efficiency but not NHEJ (Fig. 4H-K and Fig. S5A-B). Moreover, we evaluated the expression of HRR effector proteins (CtIP, RAD51, and RPA32) and NHEJ effector proteins (53BP1 and RIF1) upon CPT treatment in ccRCC cells with altered ACOX2 expression. Consistently, the expression level and foci formation of CtIP, RAD51, and RPA32 were obviously upregulated in ACOX2-KD ccRCC cells. The effects could be abrogated by re-expression of ACOX2-FL (but not the MRE11-binding-deficient ACOX2-MD1 mutant) in ACOX2-KD cells (Fig. 4L-P and Fig. S5C-G). In contrast, no significant change of 53BP1 and RIF1 was observed (Fig. 4Q-T and Fig. S5H-K). Taken together, these results suggest that ACOX2 impairs HRR and aggravates DSBs in ccRCC by destabilizing the MRN complex.

### ACOX2 activates the cGAS-STING pathway and is associated with more mature TLS

**Fig. 5:**
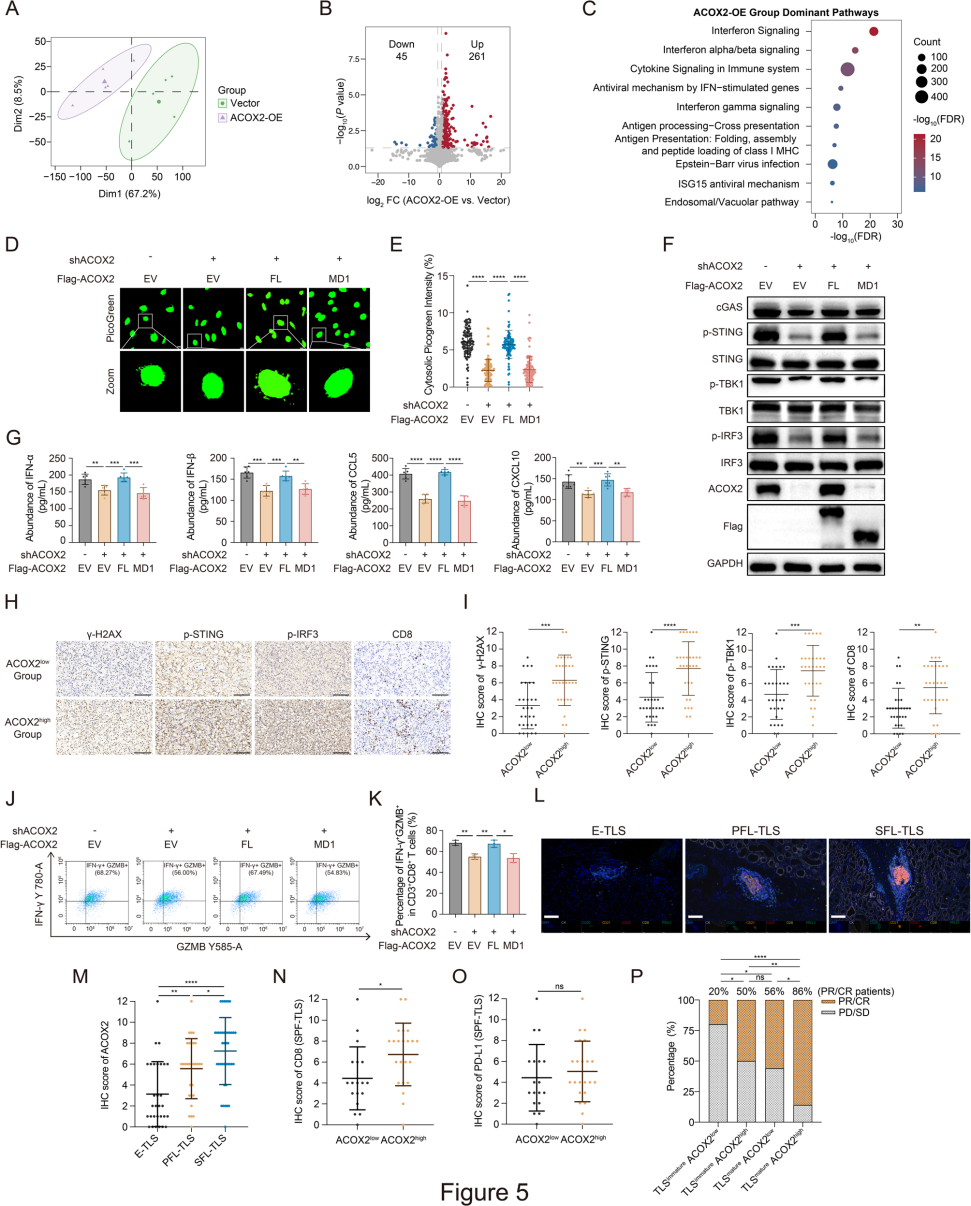
ACOX2 activates the cGAS-STING pathway and implies for mature TLS in ccRCC. **A** The PCA plot between Vector and ACOX2-OE groups. **B** The volcano plot showing DEPs in ACOX2-OE group versus Vector group. **C** The up enriched biological pathway in ACOX2-OE group versus Vector group. **D, E** Representative micrographs (**D**) and quantification data (**E**) for cytosolic double-stranded DNA (dsDNA) detected with the PicoGreen staining in the indicated 786-O cells. Scale bar: 10 μm. **F** Immunoblotting of the cGAS-STING pathway markers in the indicated 786-O cells. **G** Enzyme-linked immunosorbent assay (ELISA) of IFN-α, IFN-β, CCL5, and CXCL10 in the indicated 786-O cells. **H, I** Representative IHC micrographs (**H**) and quantification data (**I**) with ACOX2, γ-H2AX, phospho-STING, phospho-IRF3, and CD8 expression in ccRCC, respectively. Scale bar: 100 μm. **J, K** Representative flow cytometry image (**J**) and quantification data (**K**) of IFN-γ^+^GZMB^+^CD8^+^ T cells co-cultured with the indicated 786-O cells. **L** Representative multiplex immunohistochemistry (mIHC) staining for E-TLS, PFL-TLS, and SFL-TLS in postoperative ccRCC specimens. Scale bar: 100 μm. **M** Quantification of IHC staining for ACOX2 in ccRCC specimens with E-TLS, PFL-TLS, and SFL-TLS. **N, O** Quantification of IHC staining for CD8 (**N**) and PD-L1 (**O**) between ACOX2^low^ and ACOX2^high^ groups in ccRCC specimens with SPF-TLS. **P** The percentage of progressive disease (PD)/ stable disease (SD) and partial response (PR)/ complete response (CR) ccRCC patients received ICI treatment among indicated groups. Statistical significance was determined by two-tailed unpaired t-test (**E**, **G**, **I**, **K**, **M**-**O**) and two-tailed Fisher's exact test (**P**). *P < 0.05, **P< 0.01, ***P < 0.001, ****P < 0.0001, and ns *P* ≥ 0.05. Experiments were independently repeated three times with similar results; data of one representative experiment are shown (**D**, **F**, **H, J, L**)

To further investigate the role of ACOX2 in ccRCC, we conducted proteomic profiling analysis in 786-O cells (ACOX2-OE group versus Vector group) (Fig. 5A and S6A-B). Among the DEPs identified, 261 were up-regulated and 45 were down-regulated in the ACOX2-OE group compared to Vector group (Fig. 5B**)**. Pathway enrichment analysis revealed a significant association with interferon signaling upon ACOX2 upregulation (Fig. 5C). Recently, the interplay between DDR and anticancer immunity has drawn great attention, among which the cGAS-STING pathway is highlighted as the bridge from sensing DNA damage to priming T cell against tumors via type I IFN-mediated stimulation [32]. Given the challenges of immunotherapy in ccRCC, we further explored the ACOX2's impact on the cGAS-STING pathway. ACOX2-KD significantly reduced cytosolic DNA content with PicoGreen staining, a widely used fluorescent stain binding dsDNA, which could be reversed by re-expression of ACOX2-FL but not ACOX2 MD1 mutant (Fig. 5D-E and Fig. S6C-D). Furthermore, ACOX2-KD significantly decreased the phosphorylation of STING, TBK1, and IRF3; this effect was abrogated by ACOX2-FL re-expression but not by ACOX2-MD1 (Fig. 5F and Fig. S6E). We then assessed the impact of ACOX2 on innate immune cytokine expression. As anticipated, the protein secretion levels of IFN-α, IFN-β, CCL5, and CXCL10 were triggered by ACOX2 (Fig. 5G and Fig. S6F).

Additionally, IHC assay demonstrated that elevated ACOX2 was associated with DSBs accumulation, the cGAS-STING pathway activation, and CD8^+^ T cells infiltration in ccRCC clinical specimens (Fig. 5H-I). Further co-culture assays demonstrated that ACOX2 activated CD8^+^ T cells in the MRN complex-dependent manner (Fig. 5J-K and S6G). Collectively, these results indicate that ACOX2 increases cytosolic DNA amount and activates the cGAS-STING pathway.

Emerging evidence links the cGAS-STING pathway activation to TLS formation, thereby enhancing immunotherapy response [41, 42]. Based on the reports and our previous work on TLS heterogeneity in ccRCC, we explored the role of ACOX2 in TLS [34, 35]. We analyzed surgical specimens and clinical data from 100 ICI-treated ccRCC patients (30 patients with E-TLS, 30 patients with PFL-TLS, 40 patients with SFL-TLS) (Supplementary Table 5L-P). The H&E and mIHC assays confirmed the existence of three types of intratumoral TLS (Fig. 5L and S6H). ccRCC tumors harboring more mature TLS (SFL-TLS) exhibited significantly higher ACOX2 IHC score (Fig. 5M). Within the subset of ccRCC containing SFL-TLS, high ACOX2 expression strongly correlated with elevated CD8 ^+^ T cells infiltration but not PD-L1 expression (Fig. 5N-O). Importantly, patients exhibiting mature TLS and high ACOX2 expression demonstrated superior immunotherapy response (Fig. 5P). Combined with our prior reports, these results indicate that ACOX2 is correlated with more mature TLS and jointly suggest better response to ICI treatment.

### ACOX2 sensitizes ccRCC cell, CDX, PDO, and PDX to PARPi treatment

**Fig. 6:**
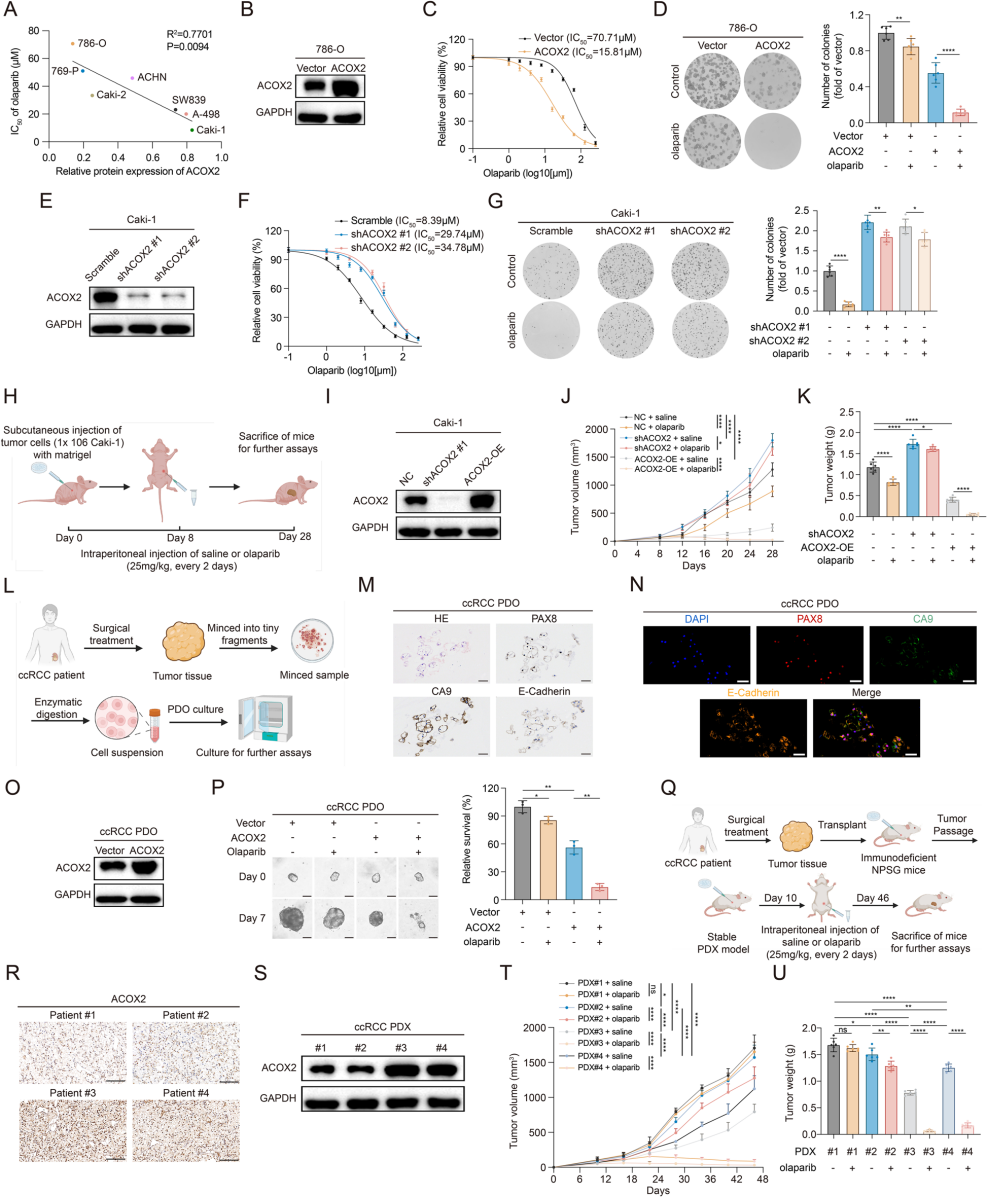
ACOX2 sensitizes ccRCC cell, cell-derived xenograft, patient-derived organoid, and patient-derived xenograft to PARPi. **A** The correlation between ACOX2 expression and half-maximal inhibitory concentration (IC_50_) of olaparib in seven RCC cell lines. **B** Immunoblotting of ACOX2 in the indicated 786-O cells. **C** IC_50_ of olaparib in the indicated 786-O cells. **D** Colony formation assay in the indicated 786-O cells in response to olaparib. **E** Immunoblotting of ACOX2 in the indicated Caki-1 cells. **F** IC_50_ of olaparib in the indicated Caki-1 cells. **G** Colony formation assay in the indicated Caki-1 cells in response to olaparib. **H** Schematic illustration for generation of Caki-1 CDX. **I** Immunoblotting of ACOX2 in the indicated Caki-1 cells. **J**,** K** Growth curves (**J**) and tumor weight (**K**) of the indicated Caki-1 PDX groups. **L** Schematic illustration for generation of ccRCC patient-derived organoid (PDO). **M** Representative hematoxylin-eosin (H&E) and IHC staining for ccRCC PDO. Scale bar: 40 μm. **N** Representative mIHC staining for ccRCC PDO. Scale bar: 50 μm. **O** Immunoblotting of ACOX2 in the indicated ccRCC PDO. **P** Relative PDO viability at the end of olaparib treatment detected by CellTiter-Glo 3D Cell Viability Assay kit. Scale bar: 50 μm. **Q** Schematic illustration for generation of ccRCC patient-derived xenograft (PDX). **R** Representative IHC staining of ACOX2 in the indicated ccRCC PDX. Scale bar: 100 μm. **S** Immunoblotting of ACOX2 in the indicated ccRCC PDX. **T**,** U** Growth curves (**T**) and tumor weight (**U**) of the indicated ccRCC PDX groups. Statistical significance was determined by pearson correlation analysis (**A**), two-tailed unpaired t-test (**D**, **G**, **K**, **P**, **U**), and two-way ANOVA (**J**, **T**). * *P* < 0.05, **P < 0.01, *** *P* < 0.001, **** *P* < 0.0001, and ns *P* ≥ 0.05. Experiments were independently repeated three times with similar results; data of one representative experiment are shown (**B**, **D**, **E**, **G**, **I**, **M**-**O**, **R**, **S**)

Given ACOX2’s role in HRR inhibition, we investigated whether ACOX2 confers synthetic lethality with PARPi in ccRCC. Consistent with this hypothesis, ACOX2 protein expression was inversely correlated with the IC_50_ of olaparib across seven RCC cell lines (Fig. 6A). Overexpression of ACOX2 in ACOX2-low-expressing cell lines (786-O and 769-P) increased sensitivity to olaparib, as shown by IC_50_ and colony formation assays (Fig. 6B-D and S7A-C). Conversely, knockdown of ACOX2 in ACOX2-high-expressing lines (Caki-1 and A-498) induced resistance to olaparib (Fig. 6E-G and S7D-F). In CDX models (ACOX2-NC, ACOX2-KD, ACOX2-OE), olaparib significantly inhibited tumor growth in ACOX2-OE tumors, while ACOX2-KD attenuated this therapeutic effect (Fig. 6H-K and S7G).

To further validate the effect of ACOX2 in PARPi response, we constructed ccRCC PDO and PDX models from fresh surgical specimens (Fig. 6L and Q). The H&E, IHC, and mIHC assays confirmed the successful establishment of PDO models (Fig. 6M-N). We found ACOX2-OE markedly promoted olaparib-killing effect in ccRCC (Fig. 6O-P). In the PDX model, we detected ACOX2 expression in four different ccRCC PDXs, two of which exhibited higher ACOX2 expression (PDX #3 and #4) (Fig. 6R-S). Notably, olaparib demonstrated significantly greater efficacy in these high-ACOX2-expressing PDXs (Fig. 6T-U and Fig. S7H). Collectively, these preclinical models demonstrate that ACOX2 markedly enhances ccRCC sensitivity to PARPi, highlighting its promising potential as a predictive biomarker for PARPi treatment in ccRCC.

### ACOX2 boosts anticancer immunity and enhances PARPi plus anti-PD-1 efficacy in vivo

**Fig. 7:**
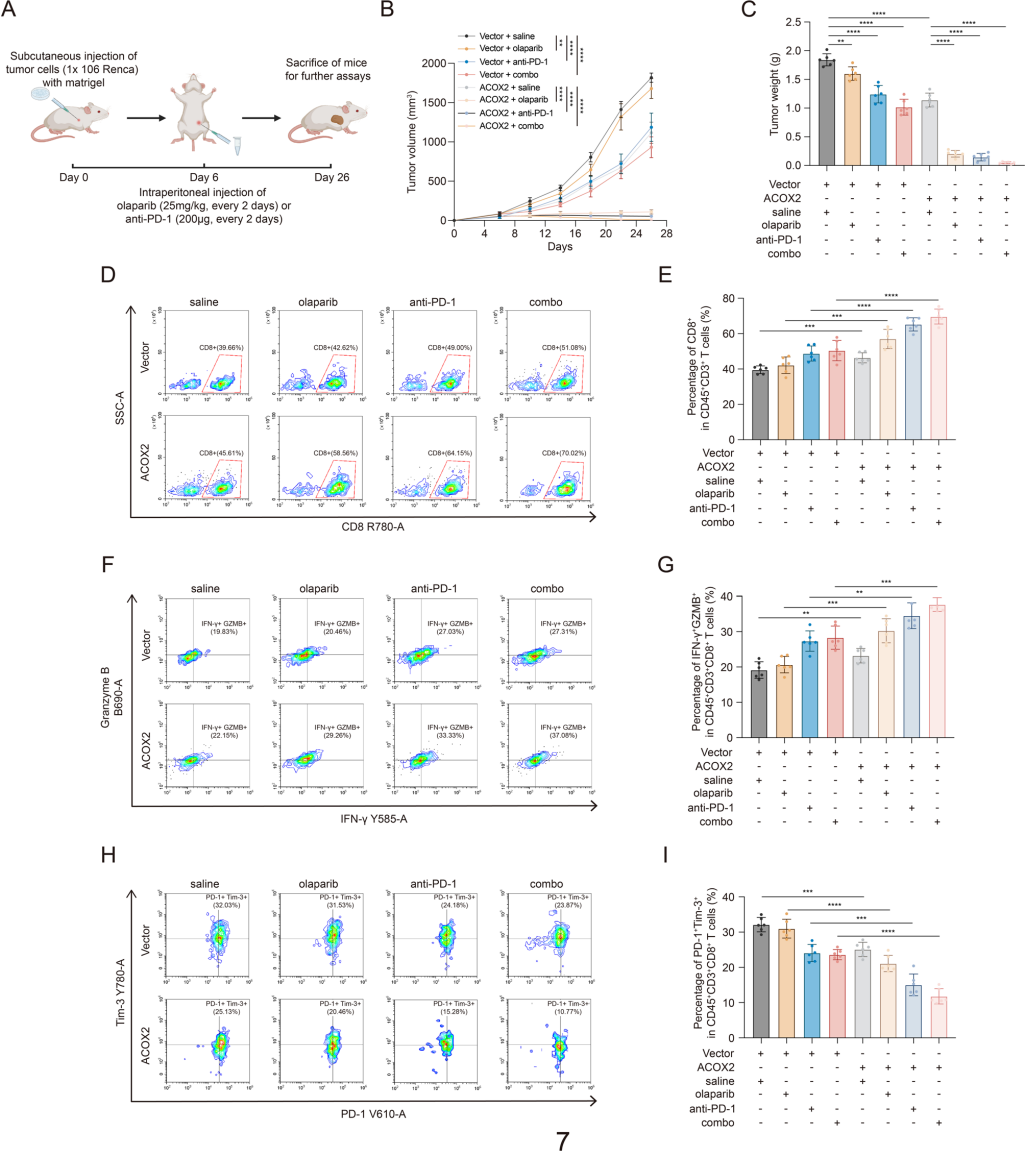
ACOX2 activates anticancer immunity and enhances anti-PD-1 plus PARPi efficacy in vivo**A** Schematic illustration for generation of immunocompetent mouse treatment model. **B**,** C** Growth curves (**B**) and tumor weight (**C**) of the indicated treatment groups. **D**,** E** Representative flow cytometry image (**D**) and quantification data (**E**) of CD8^+^ T cells (CD45^+^ CD3^+^ CD8^+^) in Renca tumors at day 26. **F**,** G** Representative flow cytometry image (**F**) and quantification data (**G**) of effector CD8^+^ T cells (CD45^+^ CD3^+^ CD8^+^ IFN-γ^+^ Granzye B^+^) in Renca tumors at day 26. **H**,** I** Representative flow cytometry image (**H**) and quantification data (**I**) of exhausted CD8^+^ T cells (CD45^+^ CD3^+^ CD8^+^ PD1^+^ Tim3^+^) in Renca tumors at day 26. Statistical significance was determined by two-way ANOVA (**B**) and two-tailed unpaired t-test (**C**, **E**, **G**, **I**). * *P* < 0.05, **P < 0.01, *** *P* < 0.001, **** *P* < 0.0001, and ns *P* ≥ 0.05. Experiments were independently repeated three times with similar results. The numbers in the graphs indicate the percentage of cells, and plots of data from one representative tumor are shown (**D**, **F**, **H**)

Building on our findings that ACOX2 activated TIME by promoting cGAS-STING pathway and correlated with TLS maturation, we evaluated its impact on immunotherapy response in vivo. Knowing that ACOX2 sensitized ccRCC to olaparib, we performed a combination treatment strategy consisting of olaparib and anti–PD-1 in immunocompetent mouse model (Fig. 7A). The results showed that ACOX2 effectively enhanced olaparib, anti–PD-1, and their combination (Fig. 7B-C and Fig. S8A). Additionally, these treatment strategies did not induce significant toxicity in mice (Fig. S8B-H).

**Fig. 8:**
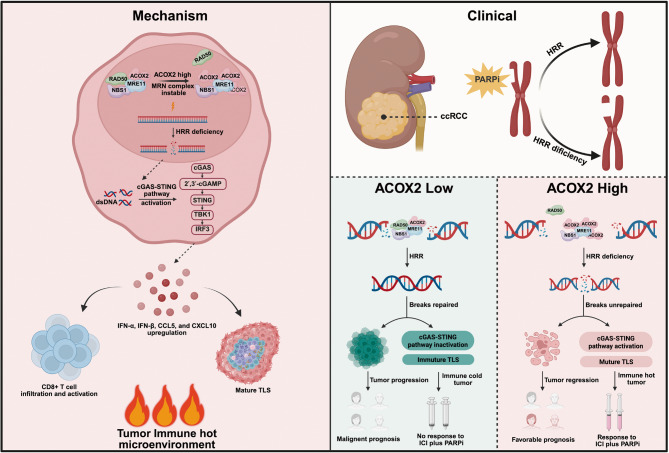
Schematic illustration of the research

As CD8^+^ T cells play a central role in anticancer immunity and are involved in the cGAS-STING pathway and the formation of TLS, we analyzed intratumoral CD8^+^ T cells populations by flow cytometry (Fig. S8I) [30, 39, 68]. ACOX2 significantly increased CD8^+^ T cells infiltration, specifically enhancing the proportion of effector CD8^+^ T cells while reducing exhausted subsets. Importantly, these effects were most pronounced in the combination treatment group (Fig. 7D-I). Collectively, these results support the therapeutic potential of combining olaparib with anti–PD-1 in ccRCC and identify ACOX2 as a promising biomarker for the treatment strategy. The overall research schematic is presented in Fig. 8.

## Discussion

The suboptimal efficacy of current immunotherapies in ccRCC underscores an urgent need for novel combination strategies and predictive biomarkers. In this research, we demonstrate a novel non-metabolic role of the classical metabolic enzyme, ACOX2, in inhibiting HRR by destabilizing the MRN complex. Critically, our findings suggest that PARPi, either alone or in combination with anti-PD-1 therapy, represent promising therapeutic approaches for ccRCC patients exhibiting high ACOX2 expression.

ACOX2, a key enzyme in peroxisomal fatty acid β-oxidation, has been increasingly recognized for its multifaceted roles in various cancers, including PCa, NSCLC, CRC, BRCA, HCC, and malignant cardiac tumor [47, 50–54]. Intriguingly, ACOX2 operates beyond its canonical metabolic function in these contexts. Our prior research demonstrated that ACOX2 regulated HCC progression via DDR pathway [47]. Similarly, we now elucidate a novel mechanism whereby ACOX2 impairs HRR in ccRCC by disrupting MRN complex assembly. These findings underscore and complement the DDR-related, non-classical tumor-regulatory role of ACOX2.

MRE11, the core component of the MRN complex, is crucial for initiating HRR and maintaining genomic stability [27]. Emerging research highlights dynamic MRE11 regulation as pivotal in cancer therapeutics [23, 27]. For example, in triple-negative breast cancer (TNBC), RNF126-mediated MRE11 ubiquitination activates the HRR and confers radiotherapy resistance [69]. In PDAC, METTL16 interacts with MRE11, inhibiting its exonuclease activity and conferring synthetic lethality to PARP inhibitors [70]. More recently, lactate-induced MRE11 lactylation in cancer cells leads to HRR hyperactivation and chemoresistance [71]. Our discovery of ACOX2-mediated MRN complex destabilization adds a significant regulatory layer to the understanding of MRE11 functionality in tumorigenesis. Besides, ACOX2 suppressed the protein expression and foci recruitment of RPA32 and RAD51 by destabilizing the MRN complex. RPA32 serves as a marker for DNA end resection, and RAD51 facilitates the formation of filaments on RPA32-coated ssDNA to mediate homology search and strand invasion [72, 73]. These results suggest that ACOX2 may impair MRE11 nuclease activity. However, further validation through direct nuclease activity assays is required to confirm this hypothesis.

The cGAS–STING pathway has garnered considerable interest in immuno-oncology due to the observation that cGAS could detect abnormal cytosolic DNA and initiate downstream immune signaling [31]. For instance, inhibiting SMYD2 in colon carcinoma mediates hypomethylation of Ku70, impairs NHEJ, and enhances antitumor immunity by activating the cGAS-STING pathway [74]. In TNBC, guanosine diphosphate–mannose (GDP-M), a tumor metabolite, suppresses HRR and triggers antitumor immunity by eliciting the cGAS-STING pathway [56]. In this study, we found that ACOX2 exacerbated DSBs and upregulated interferon-related pathways with proteomic sequencing. These findings pushed us to hypothesize that the activated cGAS-STING pathway may act as a critical bridge between these two ACOX2-regulated physiological processes. Recent work by Cho et al. demonstrated that MRE11 released cGAS from nucleosome sequestration and promoted cGAS stimulation [75]. This perspective appears to contrast with our findings. However, these observations represent complementary—not contradictory—aspects of the MRN complex’s multifaceted role in regulating cGAS-STING signaling. Cho et al. focus on MRN’s role in the nuclear compartment, which enables cGAS oligomerization and initial activation before DNA fragments reach the cytoplasm [75]. Our study reveals that destabilizing the MRN complex compromises its canonical HRR function, leading to persistent DNA damage, increasing cytosolic DNA amounts, and subsequent cytosolic cGAS sensing. Thus, we probe the consequence of MRN dysfunction on downstream cytosolic cGAS-STING activation via increased ligand availability. The MRN complex thus has dual context-dependent roles. Functional MRN complex licenses cGAS for rapid response to nuclear DNA damage. When the complex is destabilized, defective repair causes pathological cytosolic DNA accumulation, overwhelmingly activating the cGAS-STING pathway. These differing observations are integrated within the DNA damage-cGAS-STING axis and collectively underscore the therapeutic complexity of targeting this pathway [76, 77].

TLS are emerging as promising biomarker for prognosis and immunotherapy response across multiple cancers [39]. Recent studies reported that activated cGAS-STING pathway reshaped the TIME, significantly induced TLS formation, and enhanced anticancer immunity in melanoma and lung carcinoma [41, 42]. Here, ACOX2 activated the cGAS-STING pathway and downstream type I interferons. Moreover, we observed that high ACOX2 expression was associated with the presence of intratumoral mature TLS, which provided preliminary insight into the mechanisms of TLS formation and maturation in ccRCC. However, direct mechanistic validation with advanced in vivo model systems remains to be explored [39]. Notably, the combination of ACOX2 expression and TLS maturity demonstrated significant translational value for jointly predicting ICI therapeutic responsiveness in ccRCC. This biomarker strategy holds immediate potential to guide precision therapy for patients with recurrent or metastatic disease post-surgery, although validation in larger prospective cohorts receiving ICI combined regimens remains essential.

Building on our findings, the clinical translation of ACOX2 represents a promising research direction. Its crucial antitumor role in ccRCC provides a theoretical foundation for direct targeting, genetic interventions, and indirect pathway regulation (e.g., STING agonists). While therapeutic development for tumor suppressors requires careful exploration—as evidenced by ongoing efforts targeting p53 and PTEN—ACOX2 also exhibits promising value as a predictive biomarker for PARPi and ICI therapy. Realizing this potential will require establishing clinically applicable expression thresholds and developing real-time monitoring methodologies during treatment. These advances will facilitate the clinical implementation of ACOX2 within precision oncology frameworks.

## Conclusions

Collectively, this study establishes the tumor-suppressive role of ACOX2 in ccRCC, demonstrated through comprehensive in vitro and in vivo models. Mechanistically, ACOX2 interacts with MRE11 and destabilizes the MRN complex, thereby inhibiting HRR and aggravating DSBs. Furthermore, ACOX2 activates the cGAS-STING pathway, enhances CD8^+^ T cells infiltration and effector function, and correlates with more mature TLS. Therapeutically, our findings identify ACOX2 as a predictive biomarker and support that PARPi, either alone or combined with anti-PD-1 therapy, represents a promising treatment strategy for ccRCC patients exhibiting elevated ACOX2 expression.

## Supplementary Information

Supplementary Material 1. Figure S1. ACOX2 is downregulated in KIRP and KICH and associated with a poorer prognosis in ccRCC. A, B Relative mRNA expression of ACOX2 in tumor and normal tissues of KIRP (A) and KICH (B) from TCGA and GTEx databases. C, D Kaplan–Meier survival curves for progression-free survival (PFS) (C) and disease specific survival (DSS) (D) of ccRCC patients with low or high expression of ACOX2 from TCGA-KIRC cohort. Statistical significance was determined by two-tailed unpaired t-test (A, B) and the two-sided log-rank (Mantel–Cox) test (C, D). * P < 0.05, **P < 0.01, *** P < 0.001, **** P < 0.0001, and ns *P* ≥ 0.05. Figure S2. ACOX2 inhibits the tumor biological characteristics of ccRCC. A, B Immunoblotting of ACOX2 in the indicated 769-P (A) and A-498 (B) cells. C, D Colony formation assay of indicated 769-P (C) and A-498 (D) cells. E, F Growth curves of indicated 769-P (E) and A-498 (F) cells using CCK-8. G, H Wound healing assay of indicated 769-P (G) and A-498 (H) cells. Scale bar: 200μm. I, J Transwell invasive assay of indicated 769-P (I) and A-498 (J) cells. Scale bar: 200 μm. K, L Percentage of apoptosis cell of indicated 769-P (K) and A-498 (L) cells with flow cytometry analysis. M, N The tumor figure of the indicated 786-O (M) and Caki-1 (N) CDX. Statistical significance was determined by two-tailed unpaired t-test (C-L). * P< 0.05, **P < 0.01, *** P < 0.001, **** P < 0.0001, and ns *P* ≥ 0.05. Experiments were independently repeated three times with similar results; data of one representative experiment are shown (A-D, G-L). Figure S3. ACOX2 neither interacts with RAD50 or NBS1 nor affects MRN complex component expression. A Co-IP between Flag-ACOX2 and RAD50 or NBS1, with or without ACOX2 overexpression. B Immunoblotting of RAD50, NBS1, MRE11, and ACOX2 in Caki-1 cells infected with indicated lentiviruses. Experiments were independently repeated three times with similar results; data of one representative experiment are shown (A and B). Experiments were independently repeated three times with similar results; data of one representative experiment are shown (A, B). Figure S4. ACOX2 inhibits homologous recombination repair in ccRCC cell. A The indicated 769-P cells were treated with or without CPT (2 μM) at the indicated time, and cell lysates were immunoblotted with the indicated antibodies. B The indicated 769-P cells were treated with or without CPT (4 h) at the indicated concentrations, and cell lysates were immunoblotted with the indicated antibodies. C The indicated 769-P cells were treated with or without CPT (2 μM, 4 h), and cell lysates were immunoblotted with the indicated antibodies. D, E Representative micrographs (D) and quantification data (E) for γ-H2AX foci formation in the indicated 769-P cells treated with or without CPT (2 μM, 4 h). Scale bar: 10 μm. F, G Representative micrographs (F) and quantification data (G) of comet assay in the indicated 769-P cells treated with or without CPT (2 μM, 4 h). Scale bar: 20μm. Statistical significance was determined by two-tailed unpaired t-test (E, G). * P < 0.05, **P < 0.01,*** P < 0.001, **** P < 0.0001, and ns *P* ≥ 0.05. Experiments were independently repeated three times with similar results; data of one representative experiment are shown (A-C, D, F). Figure S5. ACOX2 increases double-strand breaks in ccRCC cell. A The indicated HEK293T-DR-GFP cells transfected with I-Scel plasmids for HRR detection by flow cytometry analysis. B The indicated HEK293T-EJ5-GFP cells transfected with I-Scel plasmids for NHEJ detection by flow cytometry analysis. C The indicated 769-P cells were treated with or without CPT (2 μM, 4 h), and cell lysates were immunoblotted with the indicated antibodies. D-G Representative micrographs (D) and quantification data (E-G) for CtIP, RAD51, and RPA32 foci formation in the indicated 769-P cells treated with or without CPT (2 μM, 4 h). Scale bar: 10 μm. H The indicated 769-P cells were treated with or without CPT (2 μM, 4 h), and cell lysates were immunoblotted with the indicated antibodies. I-K Representative micrographs (I) and quantification data (J, K) for 53BP1 and RIF1 foci formation in the indicated 769-P cells treated with or without CPT (2 μM, 4 h). Scale bar: 10μm. Statistical significance was determined by two-tailed unpaired t-test (E-G, J, K). * P< 0.05, **P < 0.01, *** P < 0.001, **** P < 0.0001, and ns *P* ≥ 0.05. Experiments were independently repeated three times with similar results; data of one representative experiment are shown (A-D, H, I). Figure S6. ACOX2 activates cGAS-STING pathway in ccRCC A The dynamics of protein abundance identified. Proteins are quantified as normalized iBAQ value and transformed to log10 Intensity. B The cumulative number of identified proteins in 10 cell samples. C, D Representative micrographs (C) and quantification data (D) for cytosolic dsDNA detected with the PicoGreen staining in the indicated 769-P cells. Scale bar: 10 μm. E Immunoblotting of cGAS-STING pathway markers in the indicated 769-P cells. F ELISA of IFN-α, IFN-β, CCL5, and CXCLl0 in the indicated 769-P cells. G Gating strategy for flow cytometry analysis. H Representative hematoxylin-eosin (H&E) staining for intratumoral TLS in postoperative ccRCC specimen. Scale bar: 100 μm. Statistical significance was determined by two-tailed unpaired t-test (D, F). * P< 0.05, **P < 0.01, *** P < 0.001, **** P < 0.0001, and ns *P* ≥ 0.05. Experiments were repeated three times independently with similar results; data of one representative experiment are shown (C, E). Figure S7. ACOX2 sensitizes ccRCC to olaparib in vitro and in vivo. A Immunoblotting of ACOX2 in the indicated 769-P cells. B IC50 of olaparib in the indicated 769-P cells. C Colony formation assay in the indicated 769-P cells in response to olaparib D Immunoblotting of ACOX2 in the indicated A-498 cells. E IC50 of olaparib in the indicated A-498 cells. F Colony formation assay in the indicated A-498 cells in response to olaparib. G Tumor figures of Caki-1 CDX. H Tumor figures of ccRCC PDX. Statistical significance was determined by two-tailed unpaired t-test (C, F). * P < 0.05, **P < 0.01,*** P < 0.001, **** P < 0.0001, and ns *P* ≥ 0.05. Experiments were independently repeated three times with similar results; data of one representative experiment are shown (A, C, D, F). Figure S8. PARPi plus anti-PD-1 shows greater efficacy when ACOX2 elevated without obvious hepatotoxicity and nephrotoxicity. A Tumor figures of immunocompetent mouse model. B Effects of saline, olaparib, anti-PD-1, and olaparib plus anti-PD-1 on mice body weight. C, D Effects of saline, olaparib, anti-PD-1, and olaparib plus anti-PD-1 on ALT (C) and AST (D) of mice. E, F Effects of saline, olaparib, anti-PD-1, and olaparib plus anti-PD-1 on CREA (E) and UREA (F) of mice. G, H Representative HE staining for liver (G) and kidney (H) of mice treated with saline, olaparib, anti-PD-1, and olaparib plus anti-PD-1. Scale bar: 100 μm. I Gating strategy for flow cytometry analysis. Statistical significance was determined by two-tailed unpaired t-test (B-F). * P < 0.05, **P < 0.01, *** P < 0.001, **** P< 0.0001, and ns *P* ≥ 0.05. Experiments were independently repeated three times with similar results; data of one representative experiment are shown (G, H).

Supplementary Material 2.

Supplementary Material 3.

## Data Availability

In this study, three publicly accessible databases were utilized. The transcriptome data of 30 cancer types and clinical data of ccRCC patients were sourced from TCGA database (https://www.cancer.gov/tcga). The proteome data of ccRCC patients were obtained from CPTAC database (https://proteomics.cancer.gov/programs/cptac). Additionally, we included a proteome cohort of 232 Chinese ccRCC patients that we previously reported (https://www.nature.com/articles/s41467-022-29577-x#Sec50). Other original data supporting the findings of this study were included in supplementary materials.

## Abbreviations

ACOX2: Acyl-CoA oxidase 2
ATCC: American Type Culture Collection
BRCA: Breast cancer
CASP: The Comet Assay Software Project
CCF: Cytosolic chromatin fragments
CCK-8: Cell Counting Kit-8
ccRCC: Clear cell renal cell carcinoma
CDX: Cell-derived xenograft
cGAS-STING: Cyclic guanosine monophosphate-adenosine monophosphate synthase-stimulator of interferon genes
Co-IP: Co-immunoprecipitation
CPT: Camptothecin
CPTAC: Clinical Proteomic Tumor Analysis Consortium
CRC: Colorectal cancer
DDR: DNA damage repair
DEPs: Differentially expressed proteins
DSS: Disease specific survival
ELISA: Enzyme-linked immunosorbent assay
E-TLS: Early TLS
FACS: Fluorescence activated cell sorting
FUSCC: Fudan University Shanghai Cancer Center
GDP-M: Guanosine diphosphate -mannose
DSBs: Double-strand breaks
HCC: Hepatocellular carcinoma
H&E: Hematoxylin-eosin
HRR: Homologous recombination repair
ICC: Immunocytochemistry
ICI: Immune checkpoint inhibitor
IHC: Immunohistochemistry
ISGs: IFN-stimulated genes
IFNs: Interferons
KD: Knockdown
KICH: Chromophobe renal cell carcinoma
KIRC: Clear cell renal cell carcinoma
KIRP: Papillary renal cell carcinoma
mIHC: Multiplex immunohistochemistry
MRN complex: MRE11-RAD50-NBS1 complex
MS: Mass spectrometry
NHEJ: Non-homologous end joining
NSCLC: Non-small cell lung cancer
OE: Overexpression
OS: Overall survival
PARPi: Poly (ADP-ribose) polymerase inhibitor
PCa: Prostate cancer
PDAC: Pancreatic ductal adenocarcinomas
PDO: Patient-derived organoid
PDX: Patient-derived xenograft
PFL-TLS: Primary follicle-like TLS
PFS: Progression-free survival
RCC: Renal cell carcinoma
RT-qPCR: Real-time quantitative polymerase chain reaction
SFL-TLS: Secondary follicle-like TLS
TCGA: The Cancer Genome Atlas
TIME: Tumor immune microenvironment
TLS: Tertiary lymphoid structures
TNBC: Triple-negative breast cancer
TNM: Tumor-node-metastasis
VHL: Von Hippel-Lindau
