# Supplementary figures and images for: ACOX2 destabilizes the MRE11-RAD50-NBS1 complex and boosts anticancer immunity via the cGAS-STING pathway in clear cell renal cell carcinoma
Source: Mol Cancer. 2025 Oct 21;24:263. doi: 10.1186/s12943-025-02420-9 (PMC12538886; doi:10.1186/s12943-025-02420-9)

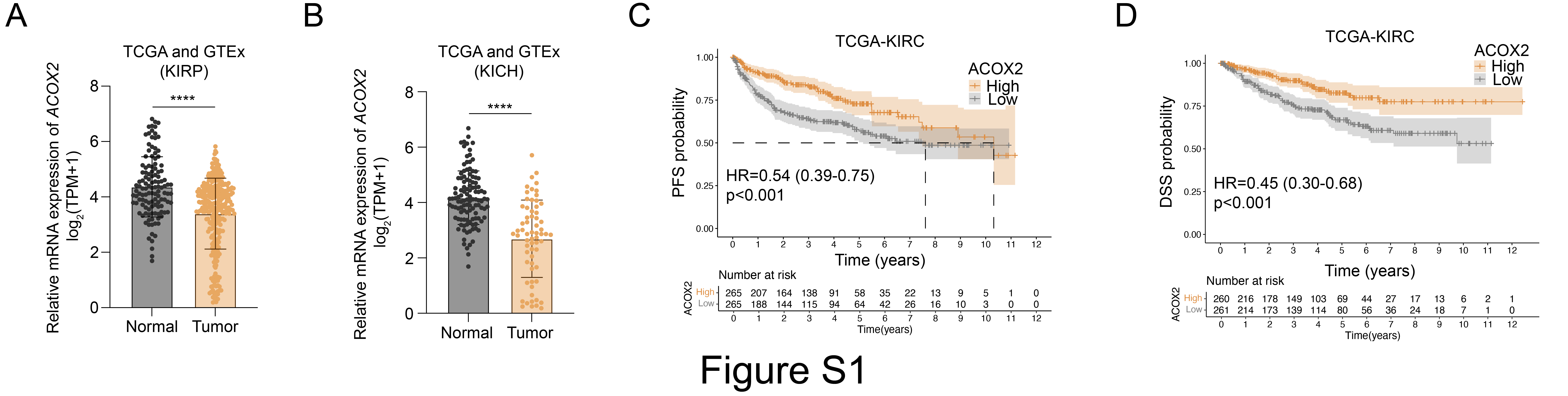

Supplement: Supplementary file 1 — Supplementary Material 1. Figure S1. ACOX2 is downregulated in KIRP and KICH and associated with a poorer prognosis in ccRCC. A, B Relative mRNA expression of ACOX2 in tumor and normal tissues of KIRP (A) and KICH (B) from TCGA and GTEx databases. C, D Kaplan–Meier survival curves for progression-free survival (PFS) (C) and disease specific survival (DSS) (D) of ccRCC patients with low or high expression of ACOX2 from TCGA-KIRC cohort. Statistical significance was determined by two-tailed unpaired t-test (A, B) and the two-sided log-rank (Mantel–Cox) test (C, D). * P < 0.05, **P < 0.01, *** P < 0.001, **** P < 0.0001, and ns P ≥ 0.05. Figure S2. ACOX2 inhibits the tumor biological characteristics of ccRCC. A, B Immunoblotting of ACOX2 in the indicated 769-P (A) and A-498 (B) cells. C, D Colony formation assay of indicated 769-P (C) and A-498 (D) cells. E, F Growth curves of indicated 769-P (E) and A-498 (F) cells using CCK-8. G, H Wound healing assay of indicated 769-P (G) and A-498 (H) cells. Scale bar: 200μm. I, J Transwell invasive assay of indicated 769-P (I) and A-498 (J) cells. Scale bar: 200 μm. K, L Percentage of apoptosis cell of indicated 769-P (K) and A-498 (L) cells with flow cytometry analysis. M, N The tumor figure of the indicated 786-O (M) and Caki-1 (N) CDX. Statistical significance was determined by two-tailed unpaired t-test (C-L). * P< 0.05, **P < 0.01, *** P < 0.001, **** P < 0.0001, and ns P ≥ 0.05. Experiments were independently repeated three times with similar results; data of one representative experiment are shown (A-D, G-L). Figure S3. ACOX2 neither interacts with RAD50 or NBS1 nor affects MRN complex component expression. A Co-IP between Flag-ACOX2 and RAD50 or NBS1, with or without ACOX2 overexpression. B Immunoblotting of RAD50, NBS1, MRE11, and ACOX2 in Caki-1 cells infected with indicated lentiviruses. Experiments were independently repeated three times with similar results; data of one representative experiment are shown (A and B). [file 12943_2025_2420_MOESM1_ESM.zip › 12943_2025_2420_MOESM1_ESM/Supplementary Figure 1.jpg]

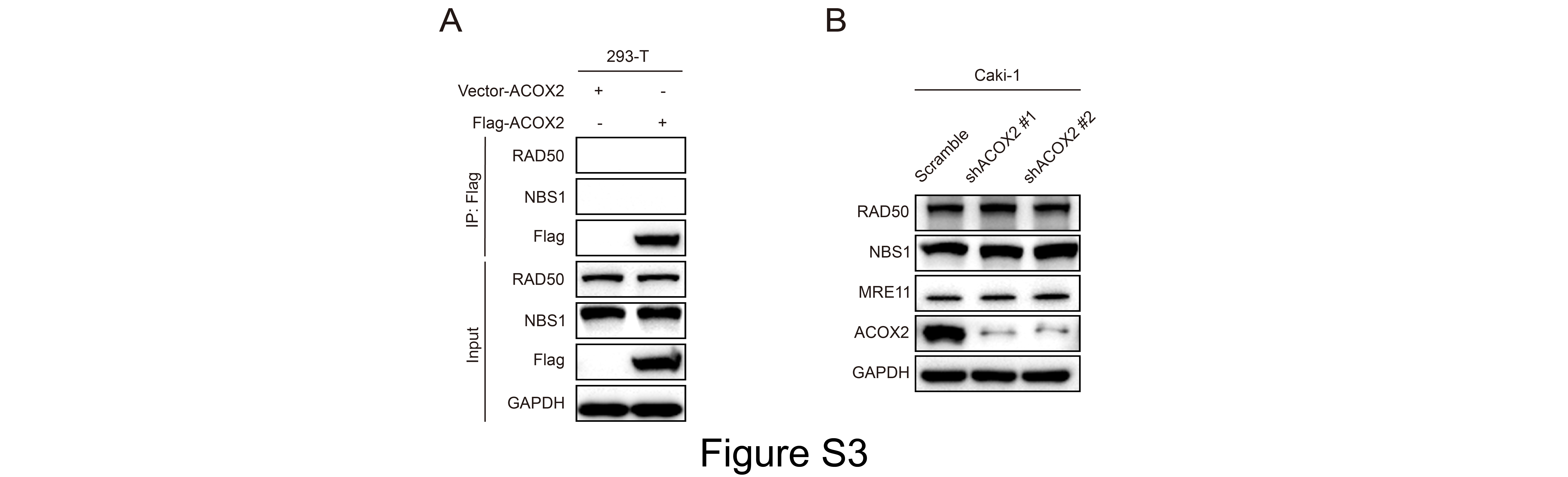

Supplement: Supplementary file 1 — Supplementary Material 1. Figure S1. ACOX2 is downregulated in KIRP and KICH and associated with a poorer prognosis in ccRCC. A, B Relative mRNA expression of ACOX2 in tumor and normal tissues of KIRP (A) and KICH (B) from TCGA and GTEx databases. C, D Kaplan–Meier survival curves for progression-free survival (PFS) (C) and disease specific survival (DSS) (D) of ccRCC patients with low or high expression of ACOX2 from TCGA-KIRC cohort. Statistical significance was determined by two-tailed unpaired t-test (A, B) and the two-sided log-rank (Mantel–Cox) test (C, D). * P < 0.05, **P < 0.01, *** P < 0.001, **** P < 0.0001, and ns P ≥ 0.05. Figure S2. ACOX2 inhibits the tumor biological characteristics of ccRCC. A, B Immunoblotting of ACOX2 in the indicated 769-P (A) and A-498 (B) cells. C, D Colony formation assay of indicated 769-P (C) and A-498 (D) cells. E, F Growth curves of indicated 769-P (E) and A-498 (F) cells using CCK-8. G, H Wound healing assay of indicated 769-P (G) and A-498 (H) cells. Scale bar: 200μm. I, J Transwell invasive assay of indicated 769-P (I) and A-498 (J) cells. Scale bar: 200 μm. K, L Percentage of apoptosis cell of indicated 769-P (K) and A-498 (L) cells with flow cytometry analysis. M, N The tumor figure of the indicated 786-O (M) and Caki-1 (N) CDX. Statistical significance was determined by two-tailed unpaired t-test (C-L). * P< 0.05, **P < 0.01, *** P < 0.001, **** P < 0.0001, and ns P ≥ 0.05. Experiments were independently repeated three times with similar results; data of one representative experiment are shown (A-D, G-L). Figure S3. ACOX2 neither interacts with RAD50 or NBS1 nor affects MRN complex component expression. A Co-IP between Flag-ACOX2 and RAD50 or NBS1, with or without ACOX2 overexpression. B Immunoblotting of RAD50, NBS1, MRE11, and ACOX2 in Caki-1 cells infected with indicated lentiviruses. Experiments were independently repeated three times with similar results; data of one representative experiment are shown (A and B). [file 12943_2025_2420_MOESM1_ESM.zip › 12943_2025_2420_MOESM1_ESM/Supplementary Figure 3.jpg]

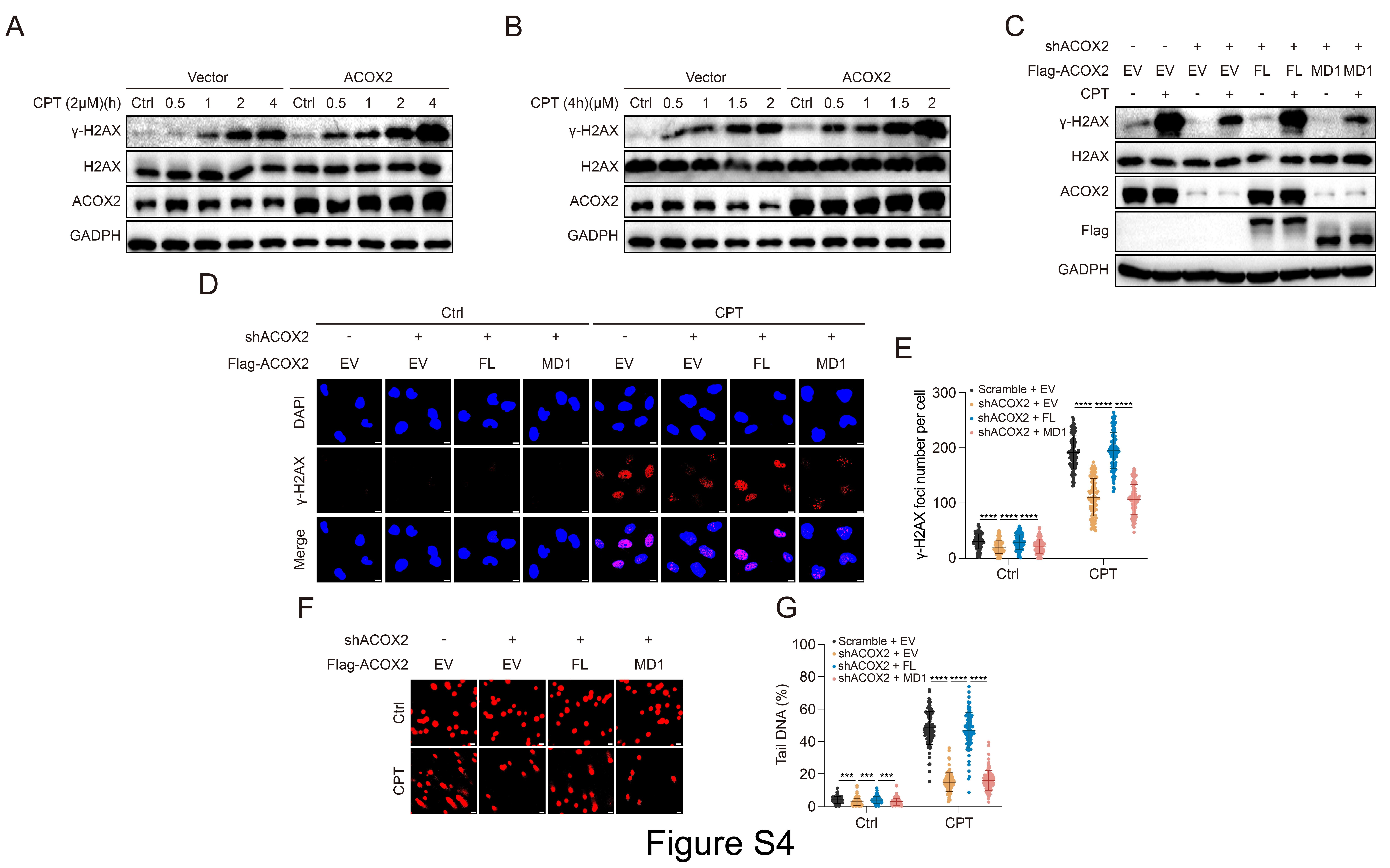

Supplement: Supplementary file 1 — Supplementary Material 1. Figure S1. ACOX2 is downregulated in KIRP and KICH and associated with a poorer prognosis in ccRCC. A, B Relative mRNA expression of ACOX2 in tumor and normal tissues of KIRP (A) and KICH (B) from TCGA and GTEx databases. C, D Kaplan–Meier survival curves for progression-free survival (PFS) (C) and disease specific survival (DSS) (D) of ccRCC patients with low or high expression of ACOX2 from TCGA-KIRC cohort. Statistical significance was determined by two-tailed unpaired t-test (A, B) and the two-sided log-rank (Mantel–Cox) test (C, D). * P < 0.05, **P < 0.01, *** P < 0.001, **** P < 0.0001, and ns P ≥ 0.05. Figure S2. ACOX2 inhibits the tumor biological characteristics of ccRCC. A, B Immunoblotting of ACOX2 in the indicated 769-P (A) and A-498 (B) cells. C, D Colony formation assay of indicated 769-P (C) and A-498 (D) cells. E, F Growth curves of indicated 769-P (E) and A-498 (F) cells using CCK-8. G, H Wound healing assay of indicated 769-P (G) and A-498 (H) cells. Scale bar: 200μm. I, J Transwell invasive assay of indicated 769-P (I) and A-498 (J) cells. Scale bar: 200 μm. K, L Percentage of apoptosis cell of indicated 769-P (K) and A-498 (L) cells with flow cytometry analysis. M, N The tumor figure of the indicated 786-O (M) and Caki-1 (N) CDX. Statistical significance was determined by two-tailed unpaired t-test (C-L). * P< 0.05, **P < 0.01, *** P < 0.001, **** P < 0.0001, and ns P ≥ 0.05. Experiments were independently repeated three times with similar results; data of one representative experiment are shown (A-D, G-L). Figure S3. ACOX2 neither interacts with RAD50 or NBS1 nor affects MRN complex component expression. A Co-IP between Flag-ACOX2 and RAD50 or NBS1, with or without ACOX2 overexpression. B Immunoblotting of RAD50, NBS1, MRE11, and ACOX2 in Caki-1 cells infected with indicated lentiviruses. Experiments were independently repeated three times with similar results; data of one representative experiment are shown (A and B). [file 12943_2025_2420_MOESM1_ESM.zip › 12943_2025_2420_MOESM1_ESM/Supplementary Figure 4.jpg]

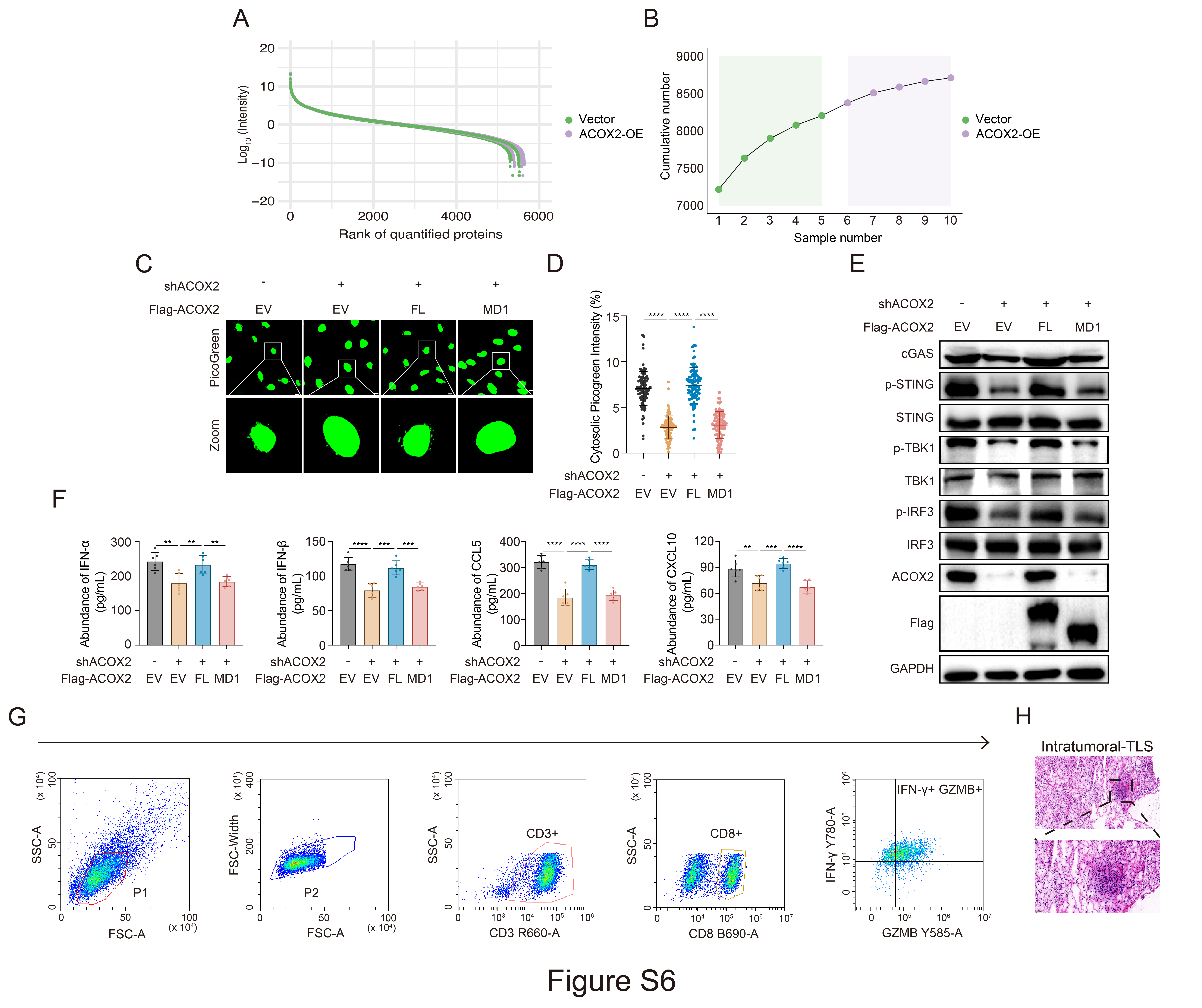

Supplement: Supplementary file 1 — Supplementary Material 1. Figure S1. ACOX2 is downregulated in KIRP and KICH and associated with a poorer prognosis in ccRCC. A, B Relative mRNA expression of ACOX2 in tumor and normal tissues of KIRP (A) and KICH (B) from TCGA and GTEx databases. C, D Kaplan–Meier survival curves for progression-free survival (PFS) (C) and disease specific survival (DSS) (D) of ccRCC patients with low or high expression of ACOX2 from TCGA-KIRC cohort. Statistical significance was determined by two-tailed unpaired t-test (A, B) and the two-sided log-rank (Mantel–Cox) test (C, D). * P < 0.05, **P < 0.01, *** P < 0.001, **** P < 0.0001, and ns P ≥ 0.05. Figure S2. ACOX2 inhibits the tumor biological characteristics of ccRCC. A, B Immunoblotting of ACOX2 in the indicated 769-P (A) and A-498 (B) cells. C, D Colony formation assay of indicated 769-P (C) and A-498 (D) cells. E, F Growth curves of indicated 769-P (E) and A-498 (F) cells using CCK-8. G, H Wound healing assay of indicated 769-P (G) and A-498 (H) cells. Scale bar: 200μm. I, J Transwell invasive assay of indicated 769-P (I) and A-498 (J) cells. Scale bar: 200 μm. K, L Percentage of apoptosis cell of indicated 769-P (K) and A-498 (L) cells with flow cytometry analysis. M, N The tumor figure of the indicated 786-O (M) and Caki-1 (N) CDX. Statistical significance was determined by two-tailed unpaired t-test (C-L). * P< 0.05, **P < 0.01, *** P < 0.001, **** P < 0.0001, and ns P ≥ 0.05. Experiments were independently repeated three times with similar results; data of one representative experiment are shown (A-D, G-L). Figure S3. ACOX2 neither interacts with RAD50 or NBS1 nor affects MRN complex component expression. A Co-IP between Flag-ACOX2 and RAD50 or NBS1, with or without ACOX2 overexpression. B Immunoblotting of RAD50, NBS1, MRE11, and ACOX2 in Caki-1 cells infected with indicated lentiviruses. Experiments were independently repeated three times with similar results; data of one representative experiment are shown (A and B). [file 12943_2025_2420_MOESM1_ESM.zip › 12943_2025_2420_MOESM1_ESM/Supplementary Figure 6.jpg]

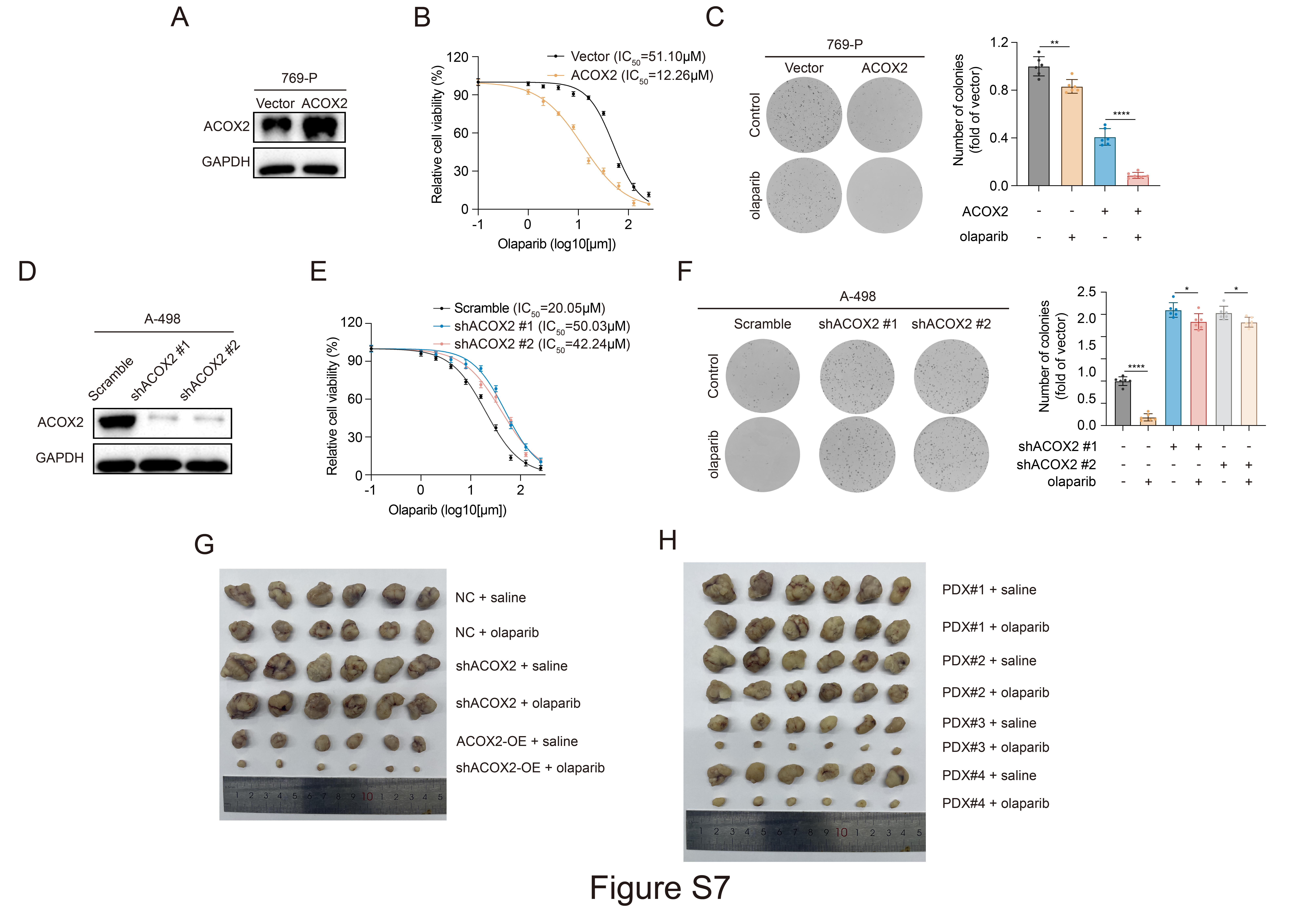

Supplement: Supplementary file 1 — Supplementary Material 1. Figure S1. ACOX2 is downregulated in KIRP and KICH and associated with a poorer prognosis in ccRCC. A, B Relative mRNA expression of ACOX2 in tumor and normal tissues of KIRP (A) and KICH (B) from TCGA and GTEx databases. C, D Kaplan–Meier survival curves for progression-free survival (PFS) (C) and disease specific survival (DSS) (D) of ccRCC patients with low or high expression of ACOX2 from TCGA-KIRC cohort. Statistical significance was determined by two-tailed unpaired t-test (A, B) and the two-sided log-rank (Mantel–Cox) test (C, D). * P < 0.05, **P < 0.01, *** P < 0.001, **** P < 0.0001, and ns P ≥ 0.05. Figure S2. ACOX2 inhibits the tumor biological characteristics of ccRCC. A, B Immunoblotting of ACOX2 in the indicated 769-P (A) and A-498 (B) cells. C, D Colony formation assay of indicated 769-P (C) and A-498 (D) cells. E, F Growth curves of indicated 769-P (E) and A-498 (F) cells using CCK-8. G, H Wound healing assay of indicated 769-P (G) and A-498 (H) cells. Scale bar: 200μm. I, J Transwell invasive assay of indicated 769-P (I) and A-498 (J) cells. Scale bar: 200 μm. K, L Percentage of apoptosis cell of indicated 769-P (K) and A-498 (L) cells with flow cytometry analysis. M, N The tumor figure of the indicated 786-O (M) and Caki-1 (N) CDX. Statistical significance was determined by two-tailed unpaired t-test (C-L). * P< 0.05, **P < 0.01, *** P < 0.001, **** P < 0.0001, and ns P ≥ 0.05. Experiments were independently repeated three times with similar results; data of one representative experiment are shown (A-D, G-L). Figure S3. ACOX2 neither interacts with RAD50 or NBS1 nor affects MRN complex component expression. A Co-IP between Flag-ACOX2 and RAD50 or NBS1, with or without ACOX2 overexpression. B Immunoblotting of RAD50, NBS1, MRE11, and ACOX2 in Caki-1 cells infected with indicated lentiviruses. Experiments were independently repeated three times with similar results; data of one representative experiment are shown (A and B). [file 12943_2025_2420_MOESM1_ESM.zip › 12943_2025_2420_MOESM1_ESM/Supplementary Figure 7.jpg]

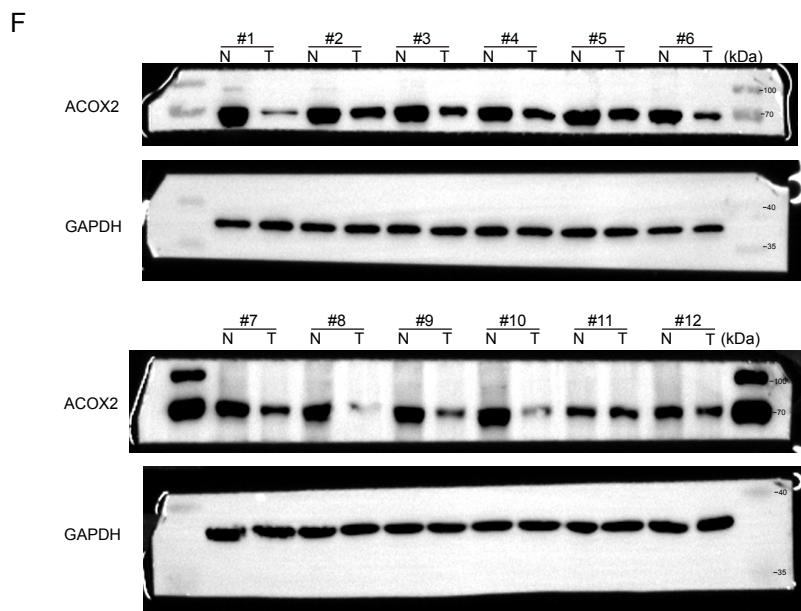

Figure 2

B

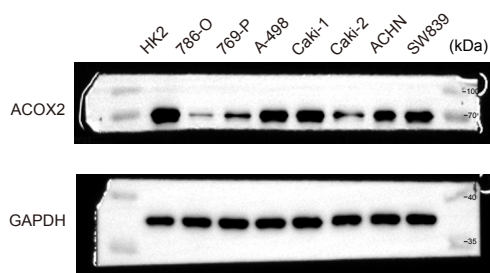

C

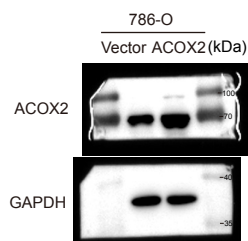

D

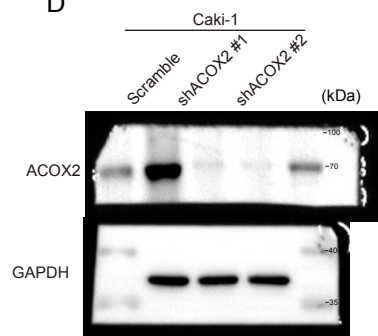

Figure 3

A

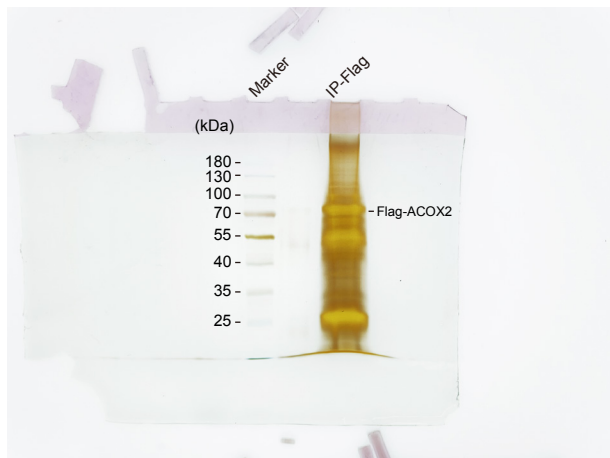

B

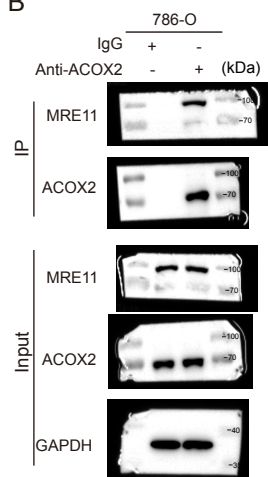

C

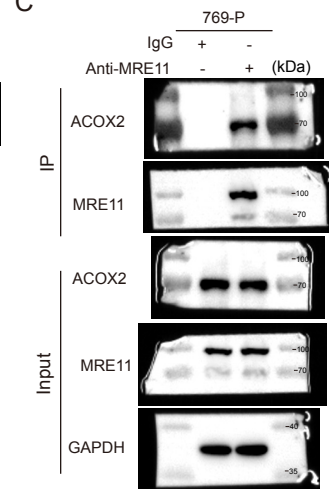

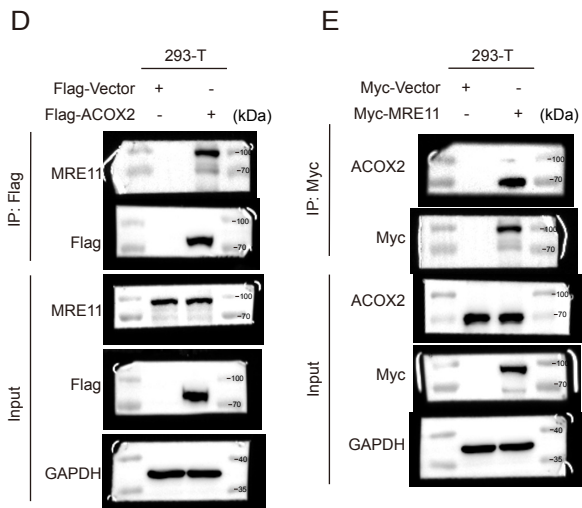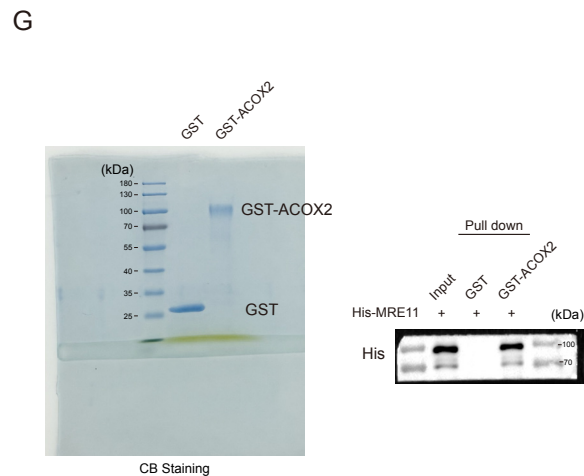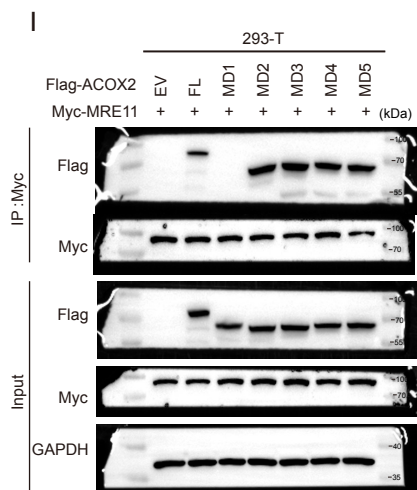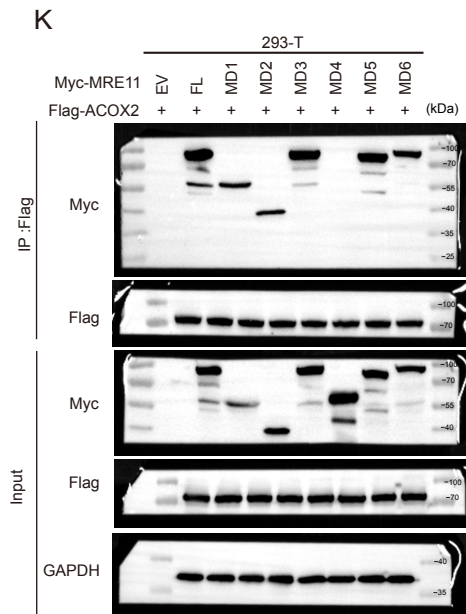

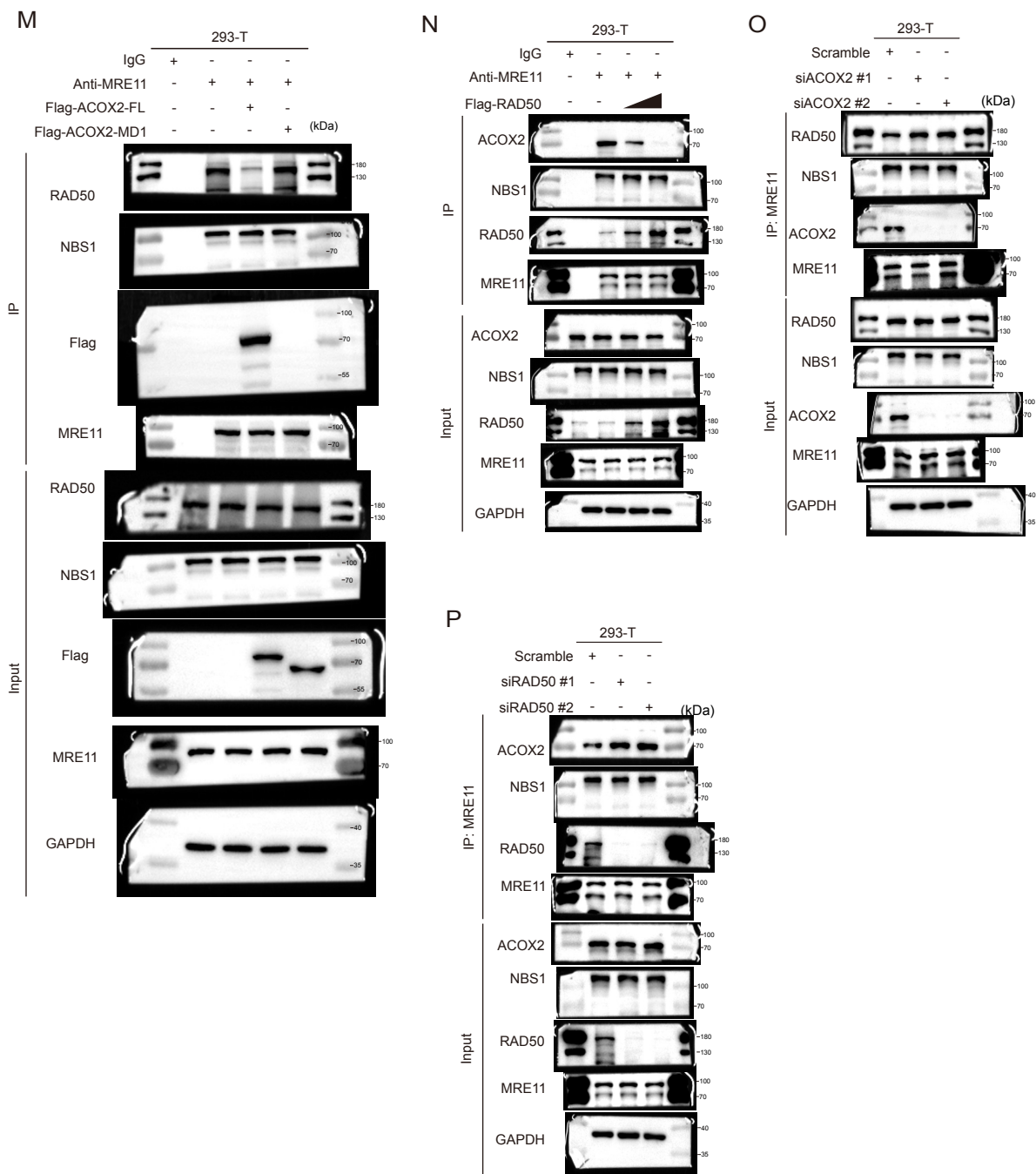

Figure 4

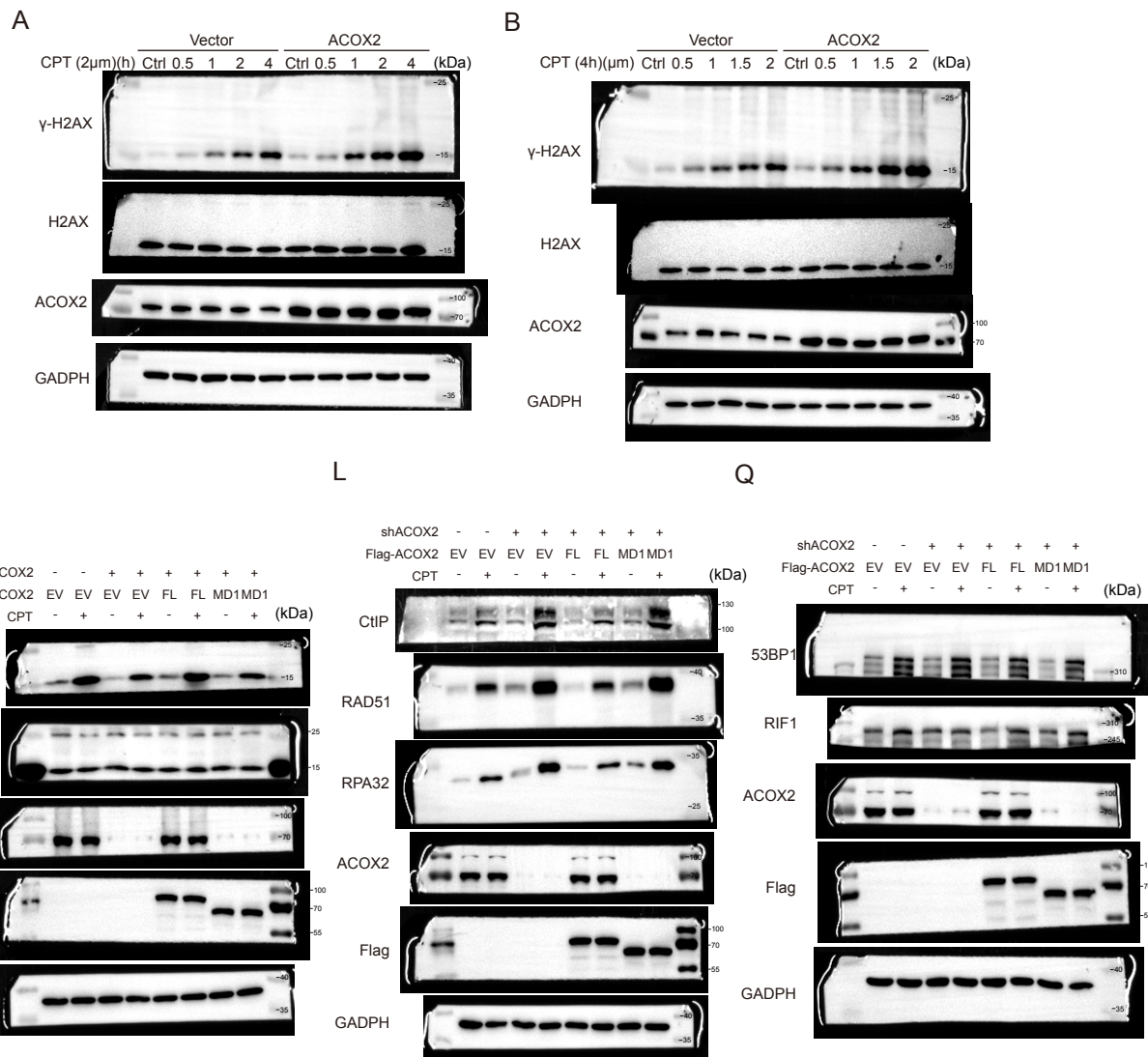

F

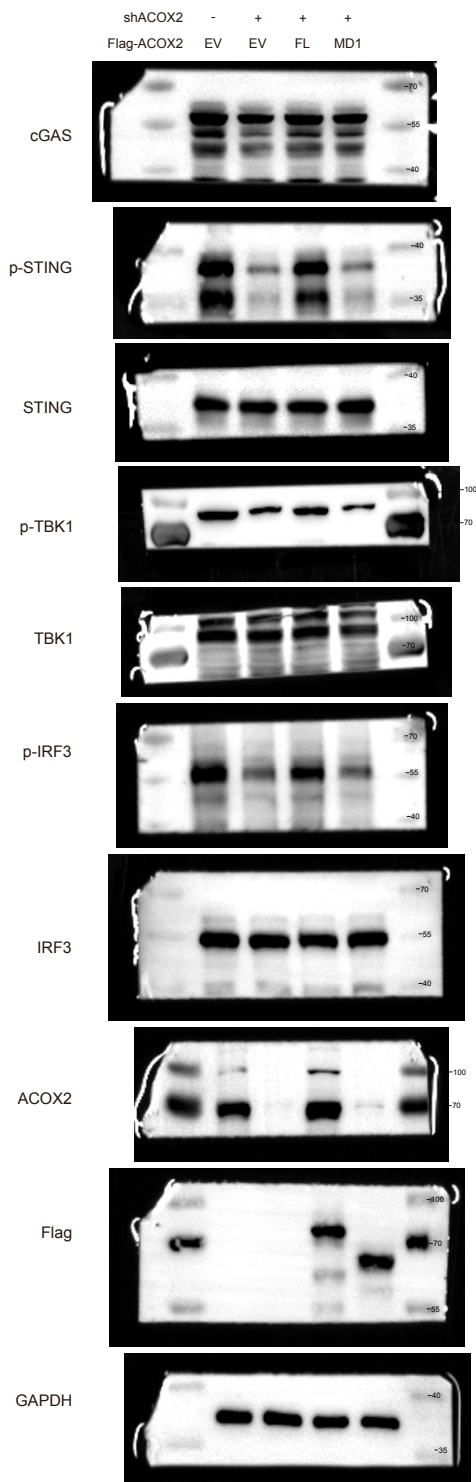

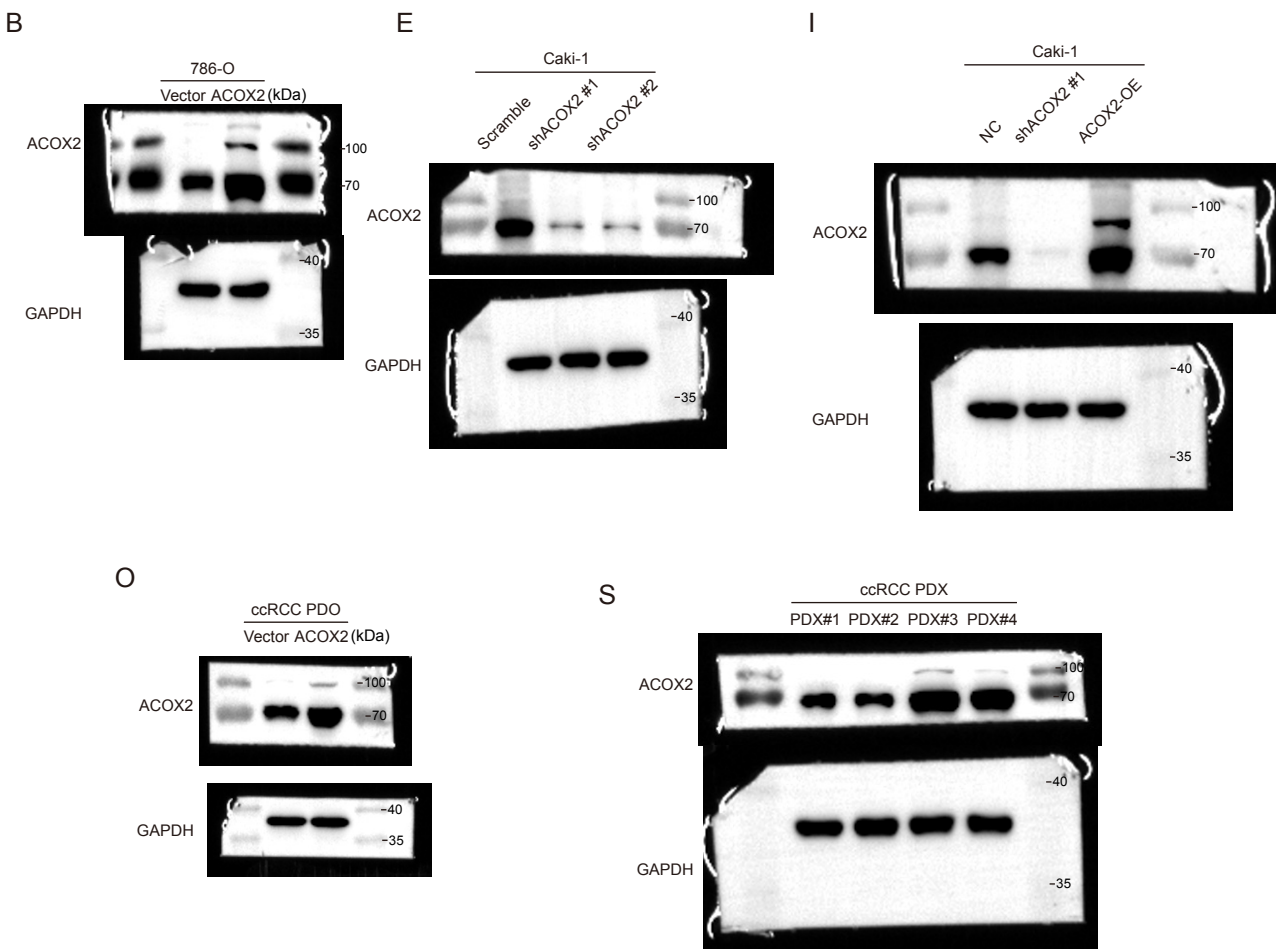

Figure S2

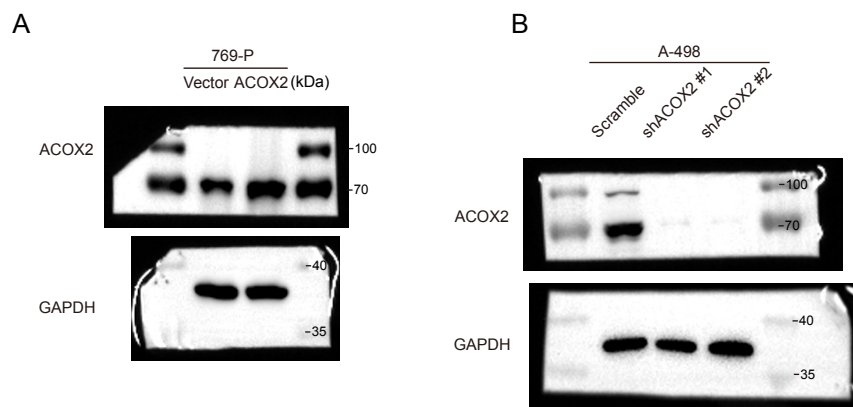

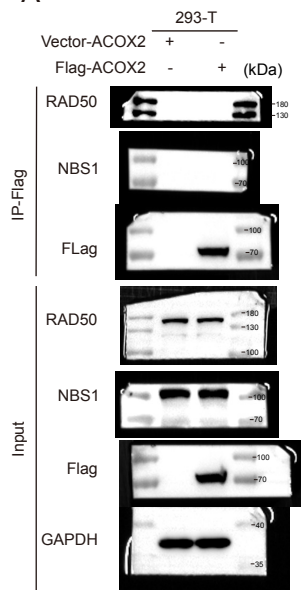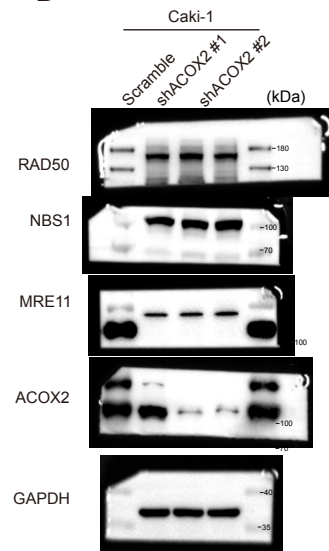

Figure S4

A

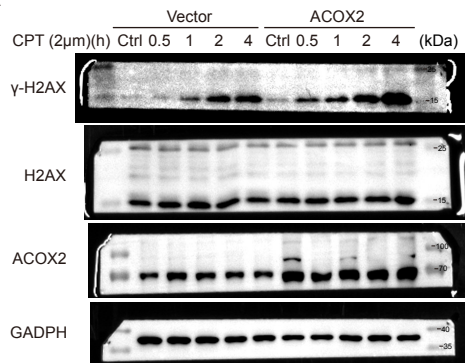

B

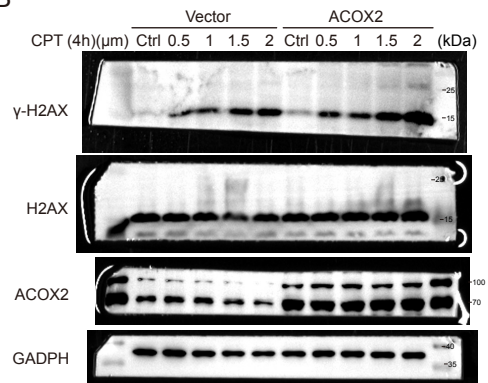

C

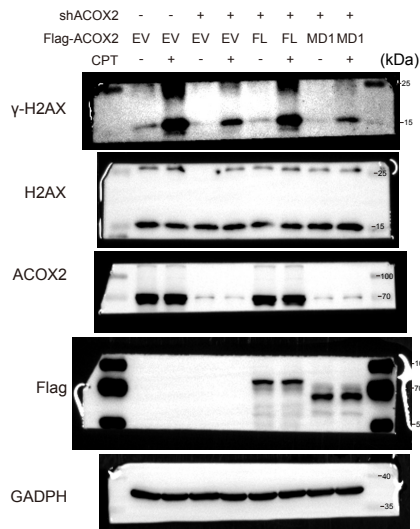

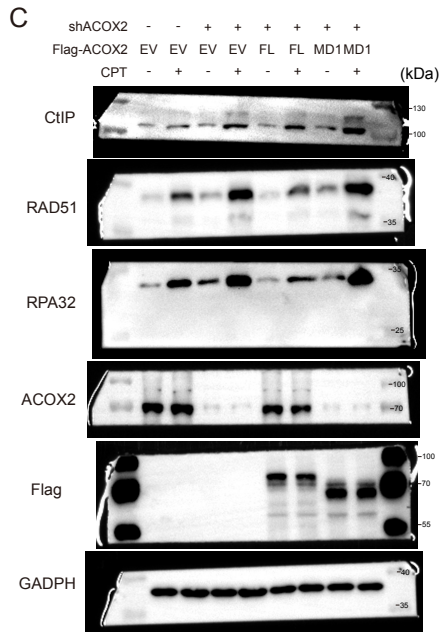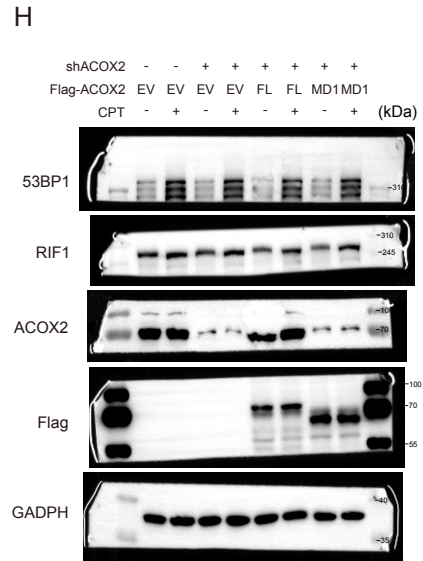

E

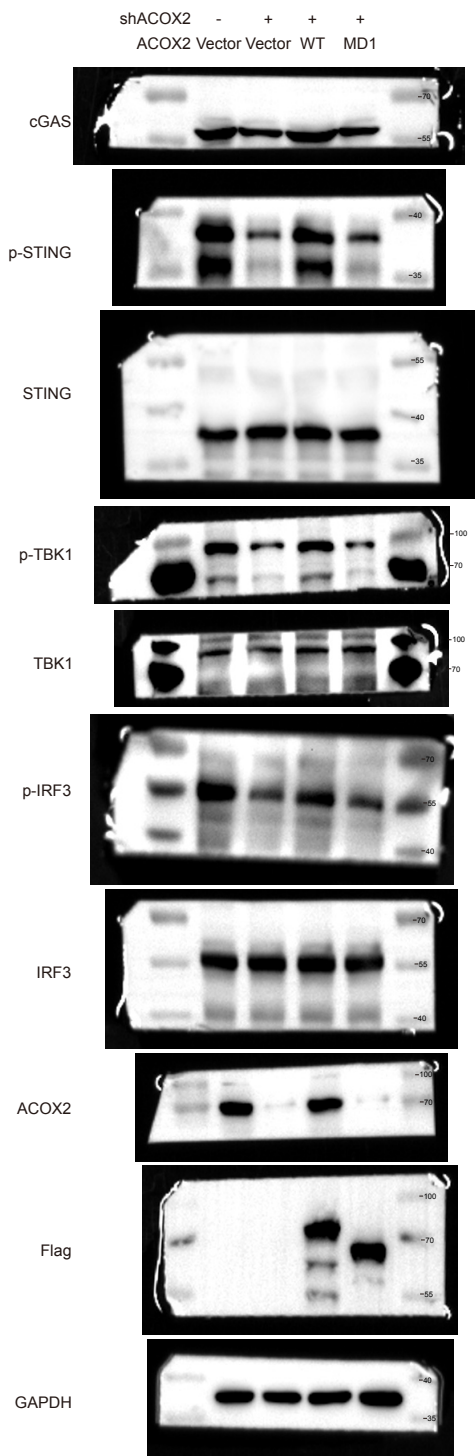

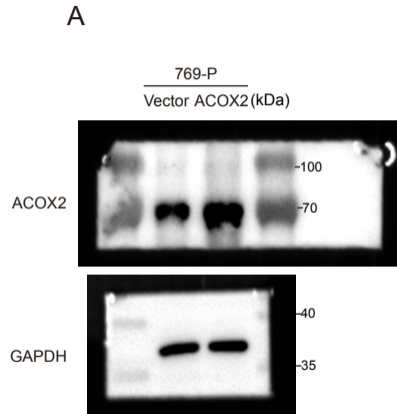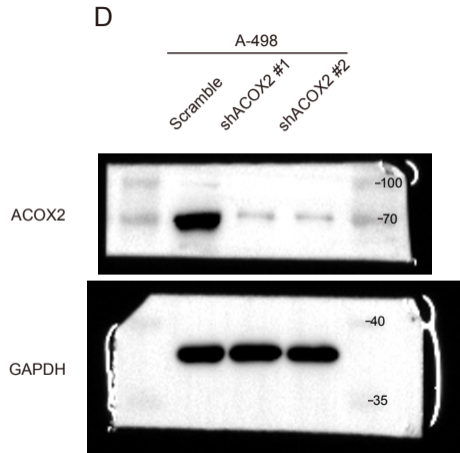

Supplement: Supplementary file 2 — Supplementary Material 2. [file 12943_2025_2420_MOESM2_ESM.pdf]
